# Synthesis and Characterization of White-Light Luminescent End-Capped Polyimides Based on FRET and Excited State Intramolecular Proton Transfer

**DOI:** 10.3390/polym13224050

**Published:** 2021-11-22

**Authors:** Atsuko Tabuchi, Teruaki Hayakawa, Shigeki Kuwata, Ryohei Ishige, Shinji Ando

**Affiliations:** 1 Department of Chemical Science & Engineering, Tokyo Institute of Technology, Ookayama 2-12-1, Meguro-ku, Tokyo 152-8552, Japan; tabuchi.a.aa@m.titech.ac.jp (A.T.); skuwata@cap.mac.titech.ac.jp (S.K.); ishige.r.aa@m.titech.ac.jp (R.I.); 2 Department of Materials & Chemical Technology, Tokyo Institute of Technology, Ookayama 2-12-1, Meguro-ku, Tokyo 152-8552, Japan; hayakawa.t.ac@m.titech.ac.jp

**Keywords:** white-light, polyimide, ESIPT, FRET

## Abstract

*N*-cyclohexylphthalimide-substituted trifluoroacetylamino (CF_3_CONH-) group (3TfAPI), which forms an intramolecular hydrogen bond, was synthesized, and it exhibited a bright yellow fluorescence owing to the excited-state intramolecular proton transfer (ESIPT) in the solution and crystalline states. In addition, CF_3_CONH-substituted phthalic anhydride (3TfAPA) was synthesized, which was attached to the termini of a blue-fluorescent semi-aromatic polyimide (PI) chain. Owing to the efficient Förster resonance energy transfer (FRET) occurring from the main chain to the termini and the suppression of deprotonation (anion formation) at the 3TfAPA moiety by H_2_SO_4_ doping, the resulting PI films display bright white fluorescence. Moreover, the enhancement of the chain rigidity by substituting the diamine moiety results in an increase in the quantum yield of white fluorescence (*Φ*) by a factor of 1.7, due to the suppression of local molecular motion. This material design strategy is promising for preparing thermally stable white-light fluorescent PIs applicable to solar spectral convertors, displays, and ICT devices.

## 1. Introduction

White light-emitting organic molecules and polymers have received a great deal of attention because they can be used for facile production of light weight, low-cost, white organic light-emitting diodes (WOLEDs), which are expected to contribute to the miniaturization of various displays and ICT devices. Moreover, solid white light-emitting organic materials based on a single luminophore typically possess high thermal and mechanical stability, as well as excellent productivity [1,2]. In general, the fluorescence spectrum of solid-state luminophores is narrow, making it difficult to obtain a luminescence spectrum covering the entire visible region. Therefore, combinations of three primary colors (red, green, and blue) or two colors (yellow/orange and blue) have been classically used to achieve white light emission [3]. However, white light generated by combining plural fluorophores often faces problems such as re-absorption, phase separation, and temporal change in emission colors. Meanwhile, white light can also be obtained from a single phase or a single molecule, by introducing two or three light-emitting elements into a single molecule [4] and single polymer [5], or using organic-inorganic hybrid semiconductors [6], metal complexes [7], and room-temperature phosphorescent polymers [8]. Achieving white light from a single-phase or molecule is beneficial because of their stability of luminescent color, ability to avoid phase separation, and simple manufacturing processes. Furthermore, when white light is emitted by exciting a single ultraviolet (UV) wavelength, the device configuration can be simplified. In any case, it is a prerequisite to achieve white-light emission to exhibit a fluorescence spectrum that covers the entire visible region.

Conventional silicon solar cells absorb little UV light because of their small bandgap, whereas they preferentially absorb long-wavelength visible light from green to red. Using fluorescent and transparent materials as a wavelength down-shifting converter integrated on a solar cell surface, the solar cell efficiency can be improved, and degradation from higher-energy UV light can be avoided [9,10,11,12,13,14]. Various types of organic dyes have been reported thus far, but many of them have problems of low photostability and an insufficient thermal stability. Due to the excellent thermal, optical, and chemical stability, fluorescent polyimides (PIs) [15] could be desirable polymeric materials for solar spectral converters because of their unique characteristics, such as excellent heat resistance, chemical stability, mechanical strength, and light resistance, originating from their rigid, repeating unit structures and strong intermolecular interactions. The fluorescent PIs can satisfy long-term use requirements even under extreme conditions, such as at elevated temperatures of >300 °C.

The electronic transitions in PI are classified into two types: ‘locally excited (LE) transition’ and ‘charge transfer (CT) transition’ [16]. The CT transition is a π−π* or n−π* transition between the highest occupied molecular orbital (HOMO) localized in the electron-donating diamine moiety and the lowest unoccupied molecular orbital (LUMO) localized in the electron-accepting dianhydride moiety of PIs. In contrast, the LE transition is a π−π* transition between molecular orbitals (MOs) localized in either the dianhydride or diamine moieties. We have analyzed the electronic transitions and fluorescence properties of fully aromatic, semi-aromatic, and fully alicyclic PIs based on time-dependent density-functional theory (TD-DFT) calculations [15]. The transition between the HOMO (S_0_) and LUMO (S_1_) of a fully aromatic PI prepared from 3,3′,4,4′-biphenyltetracarboxylic dianhydride (BPDA) and *p*-phenylenediamine is attributed to the CT(π−π*) transition, resulting in yellowish coloration and green fluorescence with a small quantum yield (*Φ*). On the one hand, the transition in a semi-aromatic PI prepared from BPDA and 4,4′-diaminocyclohexylmethane (DCHM) is attributed to the LE(π−π*) transition, resulting in colorlessness and blue fluorescence with a high *Φ*. On the other hand, that of the semi-aromatic PIs prepared from pyromellitic dianhydride (PMDA) with a short conjugation length and DCHM is attributed to the LE(n−π*) transition, exhibiting very weak fluorescence. However, since it has been reported that PI films with acid dianhydrides, such as PMDA and BPDA, emit fluorescence in the visible region, the improvement of the fluorescence property of PI is desired [16,17,18,19]. To suppress the CT and LE(n−π*) transitions and enhance the LE(π−π*) transitions between the S_0_ and S_1_ states, we have developed a series of highly fluorescent PIs with high *Φ* values by combining dianhydrides with high electron-accepting ability, whose HOMO-LUMO transitions are LE(π−π*), and alicyclic diamines with low electron-donating ability [15,20,21,22,23,24,25,26,27,28,29]. With other methods, highly fluorescent fully aromatic PIs have been developed by introducing aggregation-induced emission (AIE)-active bulky moieties, such as modified triarylamine [30,31] or tetraphenylethylene structures [32], in their main or side chains.

Conventional fluorescent PIs exhibit relatively small Stokes shifts (*ν*) because of the small energy gap between absorption and emission. Hence, white emission, which requires long-wavelength fluorescence with a very large *ν*, is challenging to achieve. To overcome this problem, the uses of excited-state intramolecular proton transfer (ESIPT) and intersystem crossing (ISC) followed by phosphorescence emission are advantageous [21,28,33]. In addition, molecules having a donor-acceptor structure exhibit large Stokes-shifted fluorescence owing to the effective spatial separation of HOMO and LUMO [34]. The Förster resonance energy transfer (FRET) is a well-known energy transfer phenomenon [35], in which the excitation energy between two chromophores in close distance is directly transferred through non-radiative dipole–dipole coupling, and has been applied to white light-emitting materials [36]. We have also reported a white-light fluorescent PI copolymer based on FRET and room-temperature phosphorescence [8]. ESIPT is a process in which the excited state is relaxed by the structural relaxation (tautomerization) from the normal (N*) form to the tautomer (T*) form, through proton transfer in the excited state, resulting in a large energy gap between the absorption and emission, leading to a very large *ν*. In particular, the ESIPT occurring at amino (-NH_2_) or substituted amino (-NHR) groups undergo more efficient proton transfer than that of the hydroxy (-OH) group, and its ESIPT reaction can be controlled by the electronegativity of the substituents [37,38,39,40]. To achieve white light with ESIPT, it is necessary to design molecules which emit yellow fluorescence via ESIPT (the emission wavelength should be around 550–580 nm) [41]. The synthesis of white light-emitting solids using ESIPT has been reported by changing the electron-donating substituents on the aromatic rings of ESIPT molecules [42], by dispersing ESIPT molecules in polymers [43], and by using nanoparticles and aggregation-induced emission (AIE) [44].

**Scheme 1:**
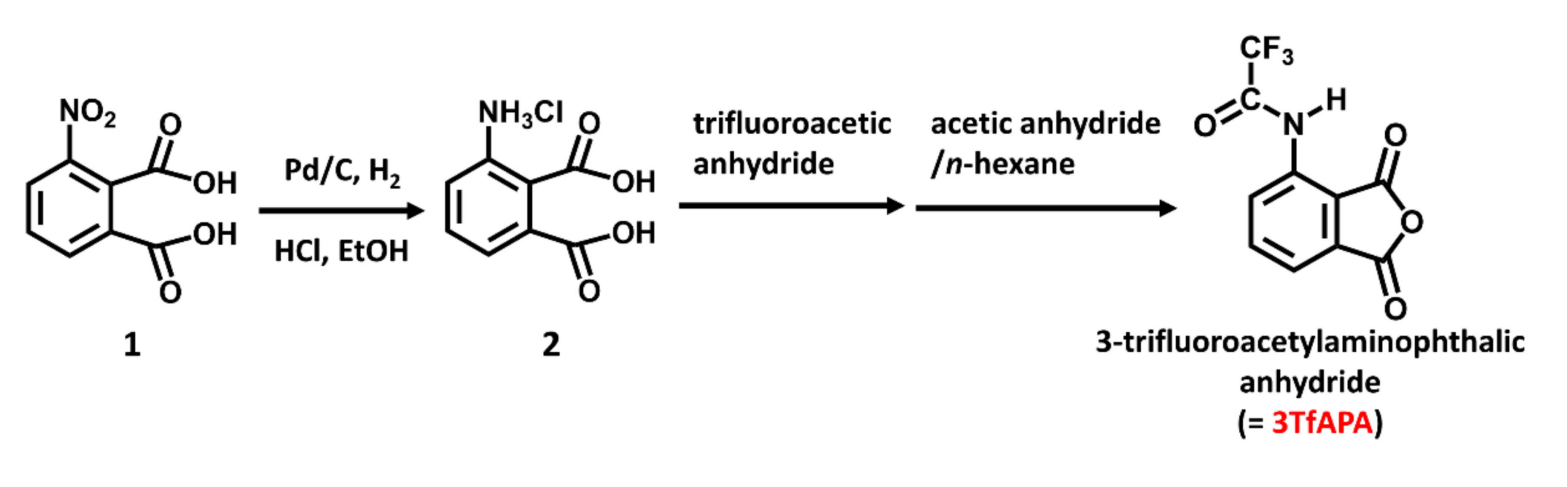
Synthesis route of 3-trifluoroacetylaminophthalic anhydride (3TfAPA).

In our previous study, a yellow fluorescent imide compound with ESIPT ability was synthesized by substituting the trifluoroacetylamino (-NHCOCF_3_) group at the 3-position of *N*-cyclohexylphthalimide (3TfAPI, Scheme S1); its fluorescent properties were briefly reported [45]. In this study, we resumed a detailed investigation of the structure and optical properties of 3TfAPI in the crystalline and solution states and we developed novel white light luminescent PI films by attaching a novel ESIPT anhydride having the same skeletal structure as 3TfAPI (3TfAPA, Scheme 1) as an end-capping agent for blue-fluorescent PIs (ODPA/DCHM or ODPA/*t*DACH).

## 2. Materials and Methods

### 2.1. Materials

From Kanto Chemical Co., Inc., 3-nitrophthalic anhydride, 3-nitrophthalic acid, and acetic anhydride were purchased and used as received. Trifluoroacetic anhydride, cyclohexylamine and Pd/C (55% water) were purchased from Tokyo Kasei Co., Ltd. and were used as received. In addition, 4,4ʹ-oxydiphthalic dianhydride (ODPA), purchased from MANAC Inc., was sublimed under reduced pressure. DCHM and *trans*-1,4-cyclohxanediamine (*t*DACH), purchased from Tokyo Kasei Co., Ltd., were purified by recrystallization from *n*-hexane and subsequent sublimation under reduced pressure. *N,O*-bis(trimethylsilyl) trifluoroacetamide (BSTFA, ≥99%) and *N,N*-dimethylacetamide (DMAc, anhydrous) were purchased from Sigma-Aldrich and used as received.

### 2.2. Preparation of Imide Compound in PMMA Matrix

The synthesis scheme and ^1^H NMR spectrum of 3TfAPI are presented in the supporting information (Scheme S1, Figure S3). The preparation scheme for the poly(methyl methacrylate) (PMMA) dispersion thin film is shown in Scheme S2. A solution was prepared by mixing 3TfAPI (1.34 × 10^−3^ g) with PMMA (0.150 g) at a concentration of approximately 1.0 wt%, using toluene (4.0 mL) as the solvent. A toluene solution containing 3TfAPI and PMMA was drop-casted onto a fused silica (amorphous SiO_2_) substrate and dried at 100 °C for 2 h under a nitrogen atmosphere. Subsequently, the film was cooled to 20 °C.

### 2.3. Synthesis of ESIPT Anhydride and Film Preparation of End-Capped Polyimides

The synthesis procedure for 3-trifluoroacetylaminophthalic anhydride (3TfAPA) is shown in Scheme 1.

Synthesis of 3-aminophthalic acid hydrochloride (**2**):

Compound **4** (4.2 g, 20 mmol) was dissolved in ethanol (84 mL), followed by the addition of 10% palladium on carbon (Pd/C, wetted with ca. 55% water) (0.94 g) and concentrated hydrochloric acid (4.2 mL). This mixture was subsequently stirred at 20 °C under a hydrogen atmosphere for 1 d. The reaction solution was passed through Celite 535 to remove Pd/C and the filtrate was concentrated using a rotary evaporator. The resulting solid was washed with acetone and dried at 50 °C for 4 h under vacuum; a white powder was obtained (yield, 92%). For ^1^H NMR (400 MHz, DMSO-*d*_6_): *δ* [ppm] 6.98 (1H, *d*, *J* = 17.2 Hz, aryl), 7.13 (1H, *d*, *J* = 18.4 Hz, aryl), 7.33 (1H, *t*, *J* = 20.8 Hz, aryl), 9.85 (2H, br *s*, NH_2_) (Figure S4).

Synthesis of 3-trifluoroacetylaminophthalic anhydride (3TfAPA):

Compound **2** (3.3 g, 15 mmol) was refluxed in trifluoroacetic anhydride (20 mL) for 3.5 h under a nitrogen atmosphere. After cooling to 20 °C, the precipitate was filtered and washed with *n*-hexane. The resulting solid was recrystallized from a mixture of acetic anhydride and *n*-hexane (acetic anhydride: *n*-hexane = 1:1), to obtain white needle-like crystals (yield 80%). For ^1^H NMR (400 MHz, Acetone-*d*_6_): *δ* [ppm] 7.94 (1H, *d*, *J* = 7.2 Hz, aryl), 8.12 (1H, *t*, *J* = 16.0 Hz, aryl), 8.61 (1H, *d*, *J* = 8.0 Hz, aryl), 10.2 (1H, br *s*, NH) (Figure S5).

**Scheme 2:**
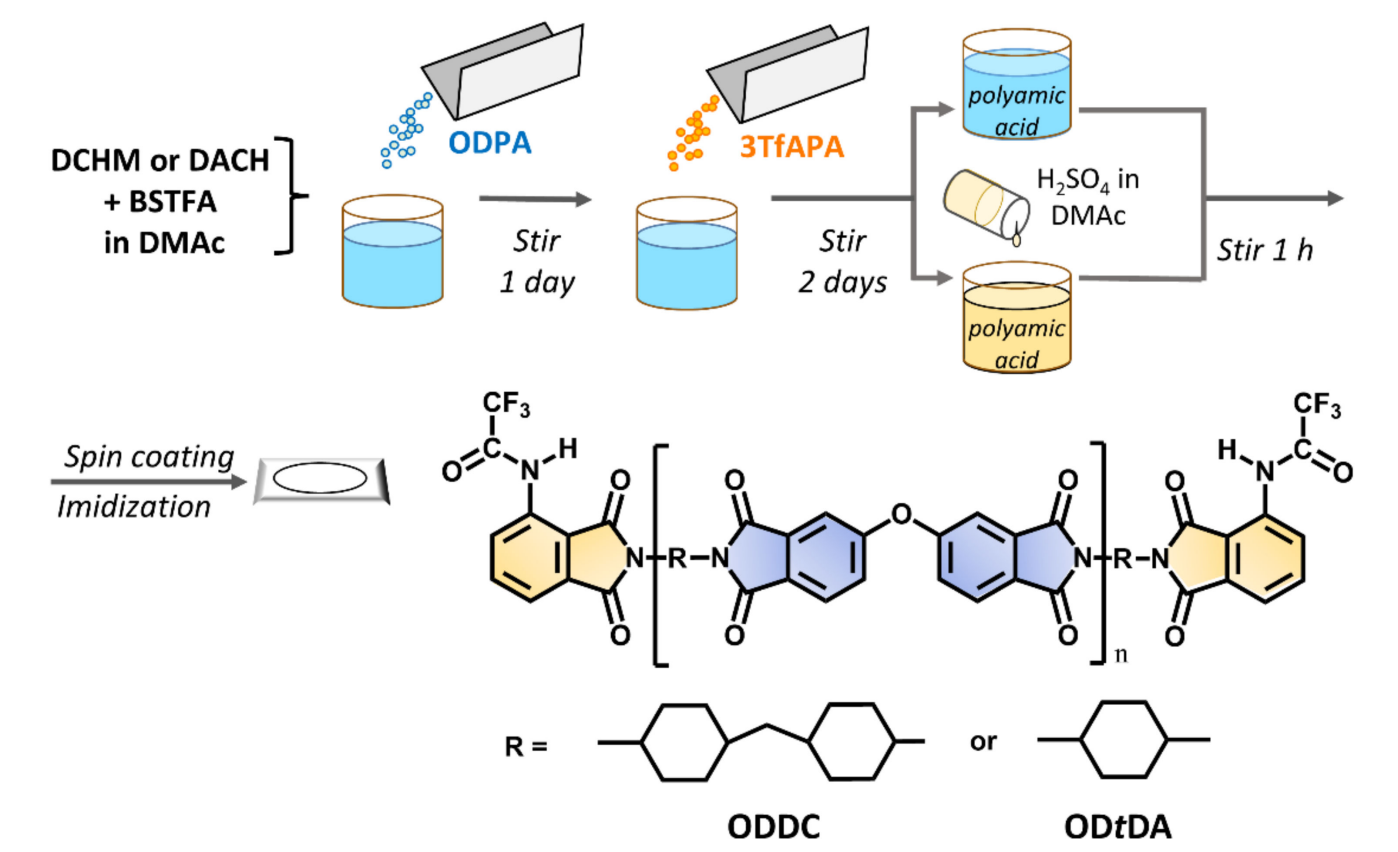
Synthesis scheme of end-capped polyimides (ODDC-TfA, OD*t*DA-TfA) with different molar ratios (*r* = 1.98, 3.96, 7.92, and 14.8).

Preparation of end-capped polyimide films:

The synthesis scheme for the end-capped PIs is shown in Scheme 2. A PI precursor, poly(amic acid) silyl ester (PASE), was prepared using an in situ silylation method reported by Matsumoto [46] and Oishi [47]. The molecular structures of the PIs of ODPA/DCHM (ODDC) and ODPA/*t*DACH (OD*t*DA) are also shown in Scheme 2. Hereafter, ODDC and OD*t*DA end-capped with 3TfAPA are abbreviated as ODDC-TfA and OD*t*DA-TfA, respectively. For example, in the case of ODDC-TfA, DCHM was dissolved in DMAc and stirred for a few minutes, then a 1.05 M amount of BSTFA was slowly added. Next, an equimolar amount of ODPA was added and stirred for 30 min at 20 °C to obtain a PASE solution. Trimethylsilylation of the amino groups of diamines by BSTFA can avoid salt formation between unreacted amino and carboxyl groups. In this case of ODDC with end groups, the molar ratio of the end group (*r*) is defined as $r=\frac{100\times m\left(\mathrm{end}\right)}{\;m\left(\mathrm{end}\right)+\;2m\left(\mathrm{ODPA}\right)}$, where *m*(end) and *m*(ODPA) are the molar quantities of the end group and dianhydride, respectively. Based on this definition, the number-average degree of polymerization (*n*) of PASE is given by (−1 + 200/*r*), with varying values of *r* = 1.98, 3.96, 7.92, and 14.8, which correspond to *n* = 100, 50, 25, and 12.5, respectively. A (100 − *r*)/100 molar amount of ODPA was then added to the trimethylsilylated DCHM solution and stirred at 20 °C for 1 d. To obtain a PASE solution, an *r*/50 molar amount of 3TfAPA was added and stirred at 20 °C for 2 days. For comparison, a PASE solution containing half or one-equimolar amounts of sulfuric acid (H_2_SO_4_) and the end group was also prepared. The anhydrides readily react with the terminal amino groups of the PASE chains, resulting in terminal-modified PASE chains. Subsequently, PI films were prepared by film-forming and thermal imidization of the corresponding precursors. The viscous and transparent PASE solution was spin-coated onto a fused silica (amorphous SiO_2_) substrate, followed by soft-baking at 70 °C for 50 min and a subsequent one-step thermal imidization until reaching the final curing conditions of 220 °C at a heating rate of 3 °C/min under a nitrogen atmosphere. The PI film was kept at 220 °C for 1.5 h and cooled to 20 °C to obtain end-capped PI films. The thicknesses of the PI films were controlled to about 2.5 µm by varying the spin-coating rate. Those of ODDC-3TfA at *r* = 1.98, 3.96 and 7.92 on fused silica were 3.6, 2.6, and 1.8 µm, those of ODDC-3TfA doped with H_2_SO_4_ (0.5 ~ 1.0 eq.) were 2.6, 3.0, and 1.6 µm, and those of OD*t*DA-TfA with *r* = 14.8 without/with H_2_SO_4_ doping (0.5 eq.) were 1.8 and 3.5 µm, respectively.

### 2.4. Measurememts

#### 2.4.1. UV–Vis Absorption and Excitation/Emission Spectroscopy

The concentration of 3TfAPI in CHCl_3_ was set to 5.0 × 10^−5^ mol/L. The solvents, chloroform (CHCl_3_, 99.0%, Kanto, FG) and trifluoroacetic acid (TFA, >99.0%, Tokyo Chemical Industry Co., Ltd., GR), were used without further purification. The UV−vis absorption and PL excitation/emission spectra were recorded separately at 20 °C using a JASCO V-760 spectrophotometer (JASCO Co., Tokyo, Japan) and a Hitachi F-7100 fluorescence spectrometer (Hitachi High-Technologies Co., Tokyo, Japan) equipped with an R928 photomultiplier tube (Hamamatsu Photonics Co., Japan), respectively. The front-face method was adopted for the film samples to reduce the self-absorption of the emitted luminescence. The spectral accuracy of this spectrometer is ±1 nm, which is larger than the wavelength resolution of 0.2 nm. Thus, the error of peak wavelengths was estimated to be less than ±1 nm.

The quantum yield of fluorescent emission (*Φ*) was measured using a calibrated integrating sphere (C9920, Hamamatsu Photonics, Hamamatsu, Japan), connected to a multichannel analyzer (C7473, Hamamatsu), and using an optical fiber link. Here, *Φ* value is an average of five measurements, and its dispersion is within ±0.002.

#### 2.4.2. Time-Resolved Luminescence Measurements

Fluorescence lifetime measurements with a low time resolution of 1 ns were conducted using a fluorescence lifetime measurement system (Quantaurus-Tau, C11367-03, Hamamatsu Photonics, Hamamatsu, Japan) at 20 °C. The decay component was recorded using excitation by applying a flashing light-emitting diode (LED) light at a wavelength of 340 nm. The fluorescence decay curves accumulated until the peak intensity reached 1000. The emission decay data were well-fitted using one to three exponentials using the deconvolution method. The lifetimes *τ*_i_ were determined as fitting parameters so that the $\chi^{2}$ value would be in the range of 1.00 ~ 1.18. Here, $\chi^{2}$ is an indicator for the degree of fitting and defined as $\chi^{2}=\sum_{j=n_{1}}^{n_{2}}\frac{{\left[I\left(t_{j}\right)-F\left(t_{j}\right)\right]}^{2}}{I\left(t_{j}\right)}/\left(n_{2}-n_{1}+1\right)$, where *I*(*t*_j_) is a calculated value for each channel, and *n*_1_ and *n*_2_ are the first and last channels in the analysis range. The residual value defined as $r\left(t_{j}\right)=\frac{I\left(t_{j}\right)-F\left(t_{j}\right)}{\sqrt{I\left(t_{j}\right)}}$ was within ±5% for all the fitting curves. Thus, the error of τ is estimated to be at most ±0.5 ns. The average lifetime was calculated as $\langle \tau \rangle =\sum_{i=1}^{n}A_{i}\tau_{i}{}^{2}/\sum_{i=1}^{n}A_{i}\tau_{i}$, where $A_{i}$ is the pre-exponential lifetime $\tau_{i}$.

#### 2.4.3. Other Measurements

^1^H NMR spectra were measured using a JEOL AL-400 spectrometer operating at a ^1^H resonance frequency of 400 MHz. The chemical shifts were calibrated in ppm (*δ*_H_) using tetramethylsilane (TMS) as the standard (0 ppm).

### 2.5. Quantum Chemical Calculations

Density functional theory (DFT) calculations were conducted using the Gaussian-16 software (Rev.C.01) [48], as described in our previous studies [49,50]. Geometry optimization was independently performed with the B3LYP and CAM-B3LYP functionals with the 6-311G(d) basis set for the ground (S_0_) and excited (S_1_) states, respectively. The 6-311++G(d, p) basis set was used to calculate the vertical excitation wavelengths and oscillator strengths (*f*) for each of the S_0_ and S_1_ geometries. The S_0_ → S_1_ transition at the optimized S_0_ geometry corresponds to the optical absorption of the ground state. In contrast, the optimized S_1_ geometry corresponds to the fluorescent emission, according to Kasha’s rule. The solvent effect of chloroform in the ground and excited states was incorporated based on the polarizable continuum model (PCM) implemented in the Gaussian software.

### 2.6. Crystallography

A needle-shaped single crystal of 3TfAPI, obtained by recrystallization from cyclohexane, was mounted on a fiber loop. Diffraction experiments were performed using a Rigaku Saturn CCD area detector with graphite-monochromated Mo-Kα radiation (*λ* = 0.71073 Å). Intensity data (6° < 2*θ* < 55°) were corrected for Lorentz-polarization effects and absorption. The structure solution and refinements were carried out using the CrystalStructure (2000–2018) program package (Rigaku Corp., Tokyo, Japan). The heavy-atom positions were determined by a direct method program (SIR92) and the remaining non-hydrogen atoms were found by subsequent Fourier syntheses and refined by full-matrix least-squares techniques against *F*^2^ using the SHELXL-2014/7 program [51]. The position of the NH hydrogen atom was refined, whereas the remaining hydrogen atoms were included in the refinements using a riding model. Crystal data: C_16_H_15_N_2_O_3_F_3_, *M* = 340.30, triclinic, *a* = 5.1357(13), *b* = 10.360(3), *c* = 14.088(4) Å, *α* = 84.565(7), *β* = 82.974(9), *γ* = 89.991(9)°, *U* = 740.5(3) Å^3^, *T* = 93 K, space group *P*$\bar{1}$ (no. 2), *Z* = 2 reflections measured, 9064 unique (*R*_int_ = 0.0584), which were used in all calculations. The final w*R*(F^2^) was 0.1007 (for all data points). CCDC 2102046 contains supplementary crystallographic data for this study.

## 3. Results and Discussion

### 3.1. Structure and Properties of Imide Compound (3TfAPI)

#### 3.1.1. TD-DFT Calculation

**Figure 1:**
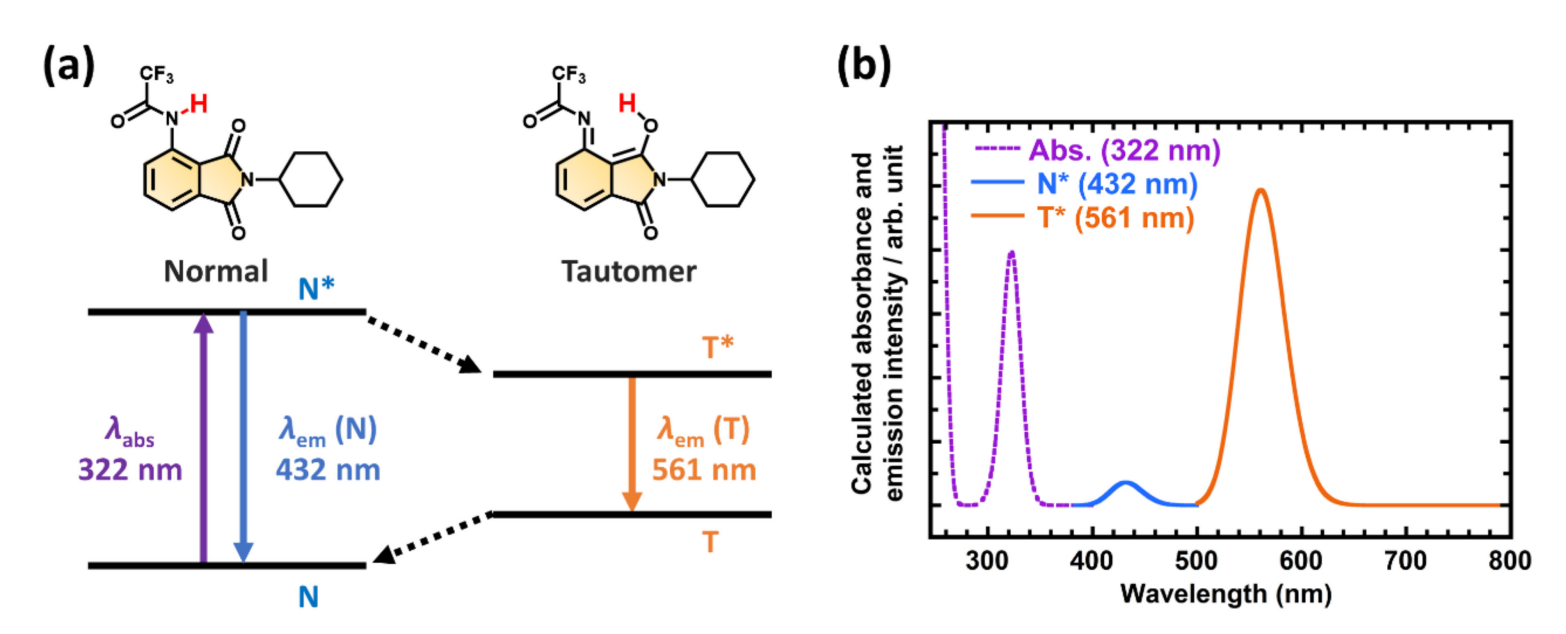
(**a**) Predicted photophysical process of the excited state intramolecular proton transfer (ESIPT) of 3TfAPI. (**b**) Calculated UV-vis absorption (dotted line) and emission (solid line) spectra of 3TfAPI in normal form (N*, blue) and tautomer form (T*, orange), under vacuum.

We have recently reported that the fluorescent color can be tuned by changing the substituent of 3-amino-*N*-cyclohexylphthalimide [45]. The targets of the molecular design for fluorophores in this study are (1) high transparency and colorlessness in the visible region and (2) intense yellowish fluorescence under UV irradiation for mixed white-color emission. Based on the TD-DFT calculations, an imide compound of 3TfAPI, in which a strong electron-withdrawing −CF_3_ group is attached to the amide linkage of the phthalimide moiety, was predicted to undergo ESIPT. In fact, it exhibited yellow fluorescence from the T* state via ESIPT, as schematically shown in Figure 1a. In addition, the calculated energy difference between the N* and T* states (*δE*_T*−N*_ = 7.35 kJ/mol) was relatively small, which indicates that the tautomerization from the N* to T* states proceeds smoothly. The calculated absorption and emission spectra of 3TfAPI are shown in Figure 1b. The lowest energy absorption of the N form and the fluorescent emission peaks of the N* and T* forms were predicted to appear at 322, 432, and 561 nm, respectively. This means that a very large *ν* (*ν* = 1.32 × 10^4^ cm^−1^) is expected for the T* emission.

#### 3.1.2. Crystal Structure

**Figure 2:**
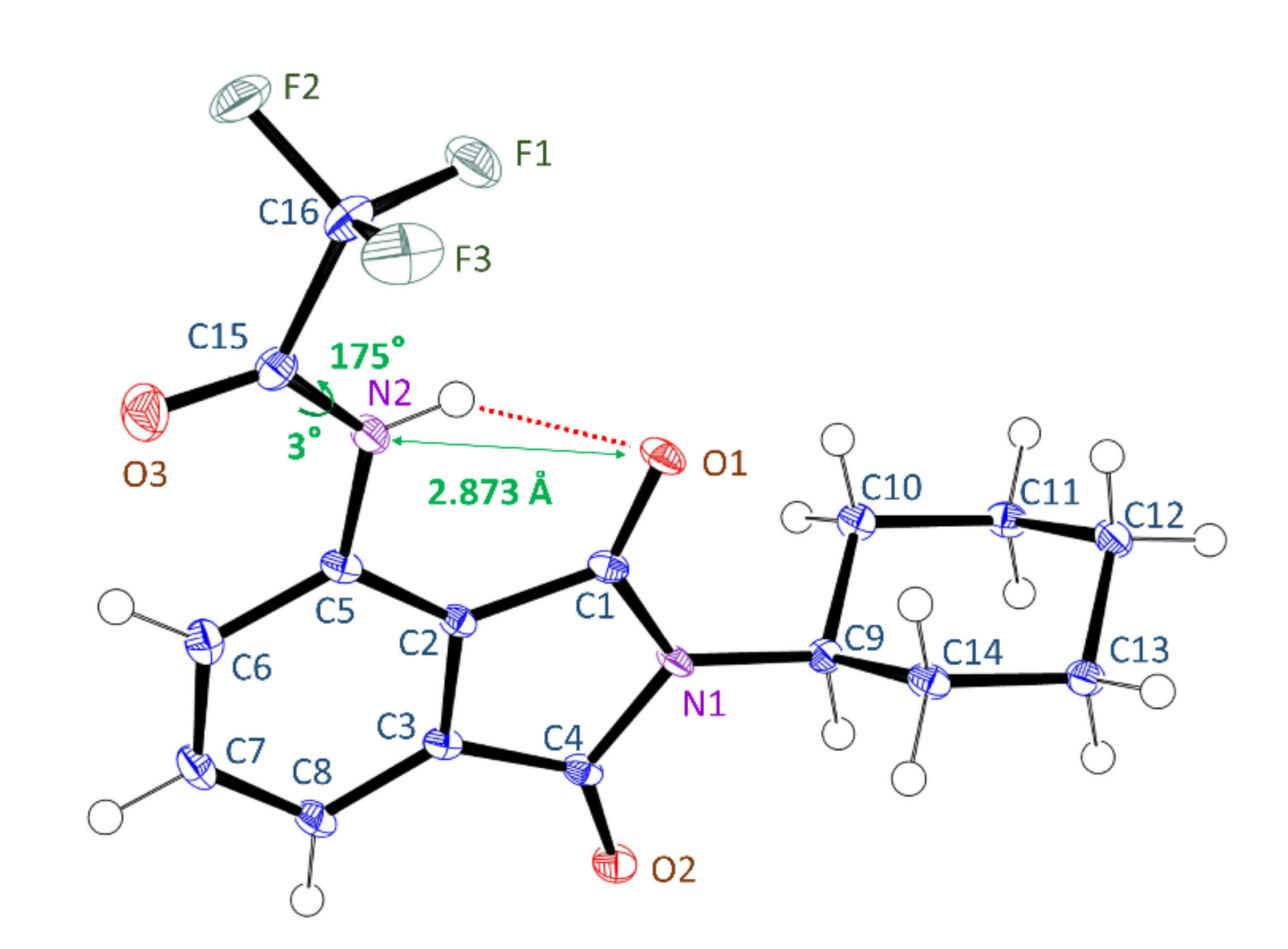
Structure of 3TfAPI in the crystal lattice determined by single-crystal X-ray diffraction analysis.

The crystal structure of 3TfAPI was determined by X-ray diffraction (XRD) analysis, as shown in Figure 2. The quality of the X-ray data was sufficient to locate the trifluoroacetamide hydrogen, which is engaged in an intramolecular hydrogen bond with one of the imide oxygen atoms (N···O, 2.873(2); NH···O, 2.19(2) Å) to form a flat, quasi-6-membered ring, which can be recognized as a resonance-assisted hydrogen bond [52]. Furthermore, the -NHCOCF_3_ group is in the same plane as the phthalimide face (C5-N2-C15-C16: 175.3(2)°, C5-N2-C15-O3: 2.6(3)°), thus 3TfAPI has an approximately symmetrical structure above and below the phthalimide face.

#### 3.1.3. Optical Properties of 3TfAPI

**Figure 3:**
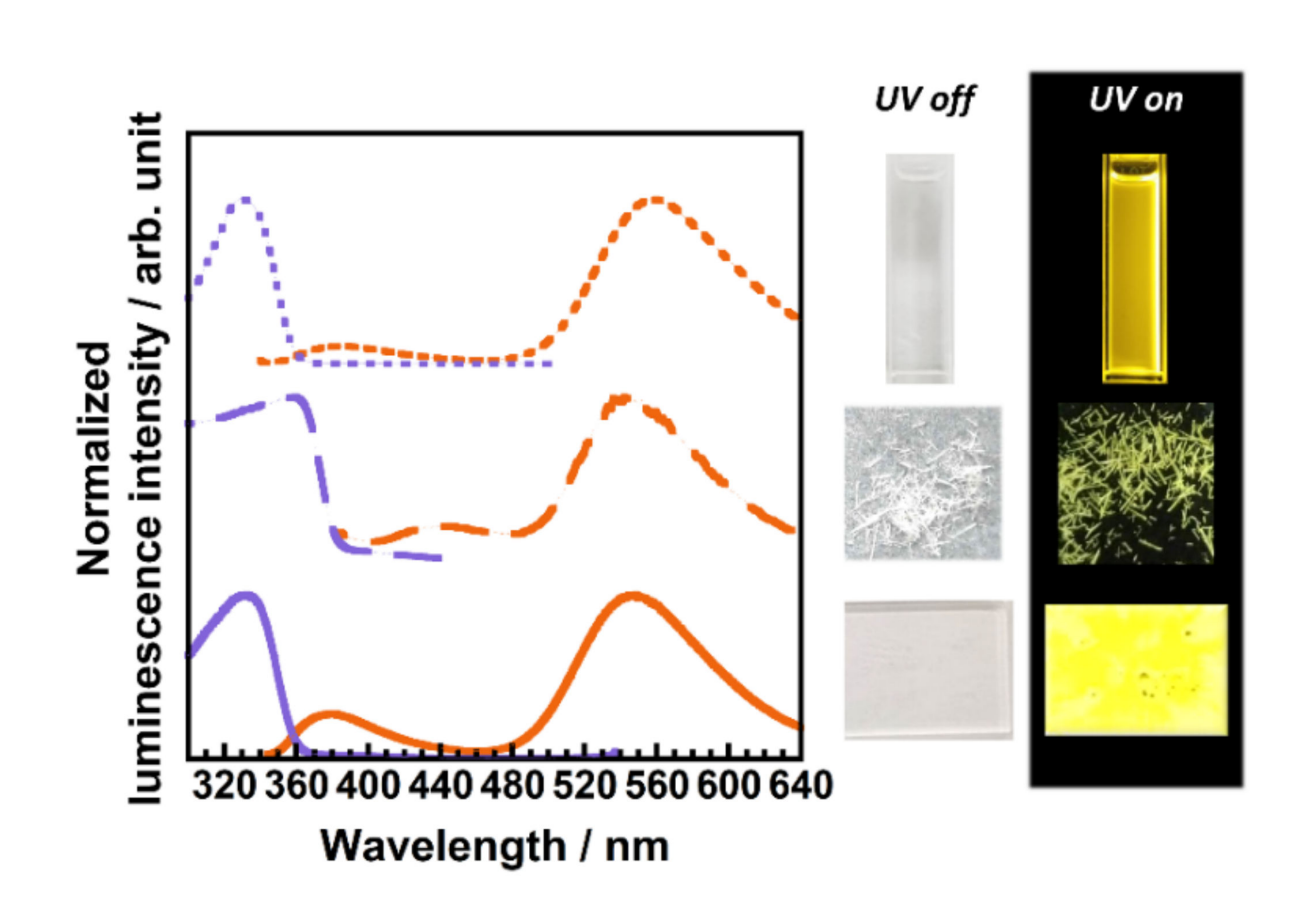
UV−vis absorption and emission spectra of 3TfAPI dissolved in CHCl_3_, in the crystalline state and dispersed in PMMA matrix with photographs of those under white light (UV off) and UV (*λ* = 365 nm) irradiation (UV on). The photograph of 3TfAPI dissolved in CHCl_3_ was taken at a concentration of 1.0 × 10^−3^ M.

Figure 3 shows the UV−vis absorption and emission spectra of 3TfAPI dissolved in CHCl_3_ (5 × 10^−5^ M), in the crystalline state, and dispersed in the PMMA matrix, along with corresponding photographs under white light (UV off) and UV irradiation of *λ* = 365 nm (UV on). In addition, the absorption and emission wavelengths (*λ*_abs_, *λ*_em_), *ν,* and *Φ* are listed in Table 1. Since the absorption edges of 3TfAPI are shorter than 400 nm, 3TfAPI is colorless under white light in all states. It is noteworthy that the fluorescence of 3TfAPI is observed at much longer wavelengths of 556, 542, and 549 nm, when excited in their respective states, at 330, 365, and 333 nm. These are readily attributable to the T* fluorescence via ESIPT because of their large *ν* (*ν* = 1.23 × 10^4^, 8.95 × 10^3^ and 1.18 × 10^4^ cm^−1^), which agrees well with the calculated wavelength of the T* fluorescence (*λ*_em_ = 561 nm, Figure 1). In contrast, the fluorescences observed in CHCl_3_ and PMMA matrix at 380 nm are attributable to the N* fluorescence, because of their small *ν* (*ν* = 3.99 × 10^3^ and 3.71 × 10^3^ cm^−1^), in accordance with the calculated wavelength of N* fluorescence (*λ*_em_ = 432 nm, Figure 1). Due to the much stronger T* fluorescence around 550 nm than N* in both states, intense yellow fluorescence was observed by UV irradiation, as shown in Figure 3.

Moreover, a high *Φ* (0.34) was observed in the crystalline state of 3TfAPI, although it decreased in the order of crystalline (0.34) > in PMMA (0.19) > in CHCl_3_ (0.17) (Table 1). The variation in *Φ* may originate from the stereochemical structure and local motion in each state. The bulky −CF_3_ substituent and the cyclohexyl group significantly reduced the intermolecular interactions and weakened the excited energy transfer through the dipolar interactions. This suppresses non-radiative deactivation, leading to a high *Φ* in the crystalline state. Meanwhile, local molecular motion of 3TfAPI is partially allowed in the PMMA matrix, and vigorous rotational and vibrational motions exist in CHCl_3_, which further reduces *Φ*.

### 3.2. End-Capped Polyimides (PIs)

#### 3.2.1. Optical Properties of ODDC-TfA

**Figure 4:**
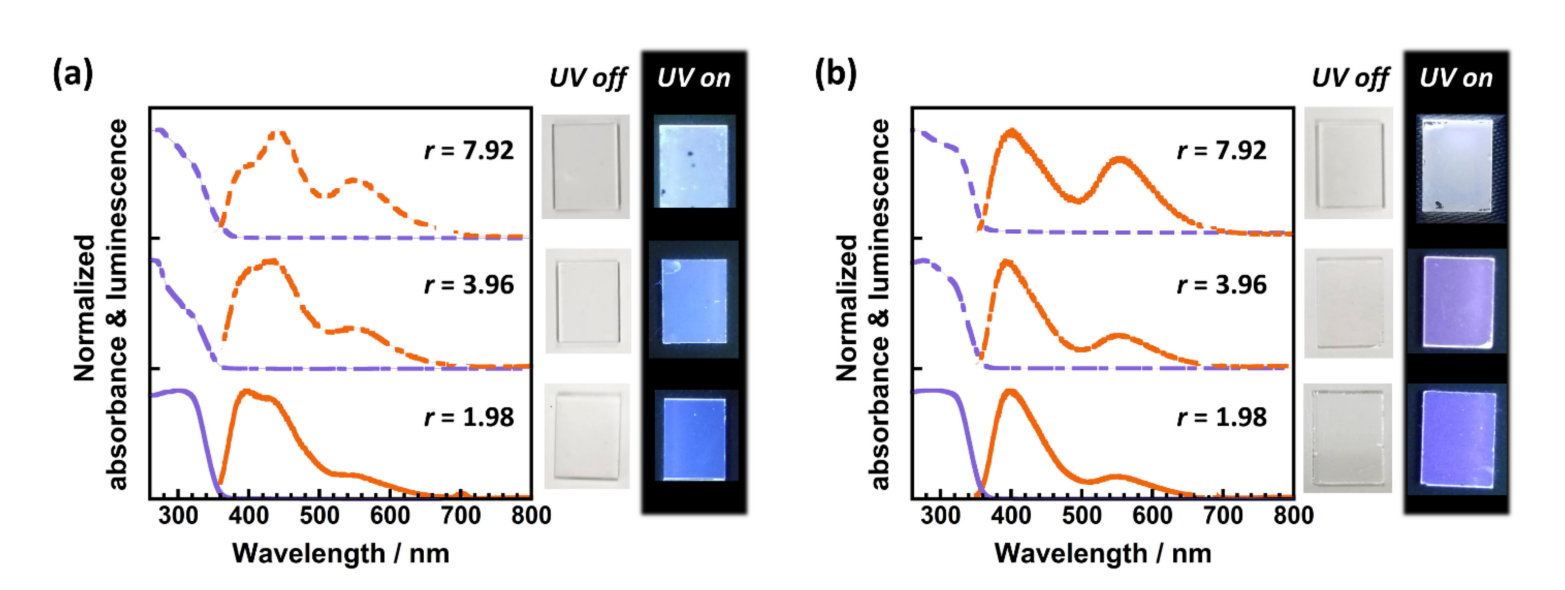
UV−vis absorption and emission spectra of (**a**) ODDC-TfA and (**b**) ODDC-TfA doped with H_2_SO_4_ (0.5 ~ 1.0 eq.), which were prepared with variable *r* values, and photographs of these films under white light (UV off) and UV (*λ* = 365 nm) irradiation (UV on).

In order to develop a colorless, flexible, and durable PI film which exhibits white-light fluorescence under UV irradiation, an end-capped PI was designed by introducing a yellow fluorescent moiety into the termini of a blue fluorescent PI, ODDC. First, a yellow fluorescent anhydride (3TfAPA) with the same skeletal structure as 3TfAPI was designed. This synthesis procedure is illustrated in Scheme 1. By passing through 3-aminophthalic acid hydrochloride (**2**), side reactions between the amino and carboxyl groups [53] were effectively suppressed, and **2** was obtained in high yield by avoiding the generation of unstable 3-aminophthalic acid. Figure 4a shows the UV-vis absorption and emission spectra of the end-capped ODDC-TfA films prepared with varying *r* values, along with the photographs under white light and UV irradiation. The experimental values of *λ*_abs_, *λ*_em_, *ν,* and *Φ* are listed in Table 2. The absorption edges of ODDC-TfA are also shorter than 400 nm, exhibiting colorless and high optical transparency under white light, and emitting bright blue to light blue fluorescence under UV irradiation (Figure 4a). It is intriguing that three emission peaks were observed at 400, 440, and 550 nm. Since the emission intensity at 400 nm decreases with increasing *r*, this fluorescence should originate from ODDC. In contrast, the emission intensities at 440 nm and 550 nm significantly increased with increasing *r*, indicating that these emissions originate from the termini end-capped by 3TfAPA. Time-resolved fluorescence decay curves are shown in Figure S6. The FRET efficiency (*E*_FRET_) can be estimated using Equation (1):

(1) $$E_{\mathrm{FRET}}=\frac{\tau_{\mathrm{ODDC}}-\tau_{\mathrm{ODDC}-\mathrm{TfA}}}{\tau_{\mathrm{ODDC}}},$$

where *τ*_ODDC_ and *τ*_ODDC-TfA_ are the fluorescence lifetimes of ODDC and ODDC-TfA at 400 nm, respectively. As listed in Table 2, the *E*_FRET_ of ODDC-TfA increased by 24%, with an increase in *r* from 1.98 to 7.92, clearly indicating that an efficient FRET process occurs from the ODDC main chain to the 3TfAPA termini within the PI. By increasing the concentration of termini, the distance between the donor (ODDC) and acceptor (3TfAPA) can be shortened, facilitating FRET. In addition, the *Φ* value slightly increases with an increase in *r*, which could be due to the suppression of local motion by the bulky 3TfAPA. In accordance with the case of 3TfAPI in Figure 3, the emission peak at 550 nm can be readily attributed to the T* fluorescence of 3TfAPA via ESIPT, as supported by the large *ν* (1.04 × 10^4^ cm^−1^ < *ν* < 1.13 × 10^4^ cm^−1^).

In our previous studies [21,45], we reported that ESIPT molecules are sensitive to the surrounding environment and may induce dissociation of the hydrogen atom in the amide group, generating an anionic form. The formation of anions in organic solvents was effectively suppressed with the addition of a small amount of TFA. Figure 4b shows the UV−vis absorption and emission spectra of ODDC-TfA doped with 0.5 ~ 1.0 eq. of H_2_SO_4_ with variable *r* values, along with photographs under white light and UV irradiation. When a small amount of non-volatile H_2_SO_4_ was added to the PAA (H_2_SO_4_-doped ODDC-TfA), the fluorescence at 440 nm was effectively suppressed [21], indicating that this fluorescence is attributable to the deprotonated anionic form, as shown in Figure S7. The *E*_FRET_ of the H_2_SO_4_-doped ODDC-TfA increased by 9% with an increase in *r* from 1.98 to 7.92, which is higher than that of ODDC-TfA. This indicates that the energy transfer from ODDC to 3TfAPA is more efficient than from the anionic form. In addition, the *Φ* values of the H_2_SO_4_-doped ODDC-TfA increased slightly with an increase in *r*, as well as ODDC-TfA.

**Figure 5:**
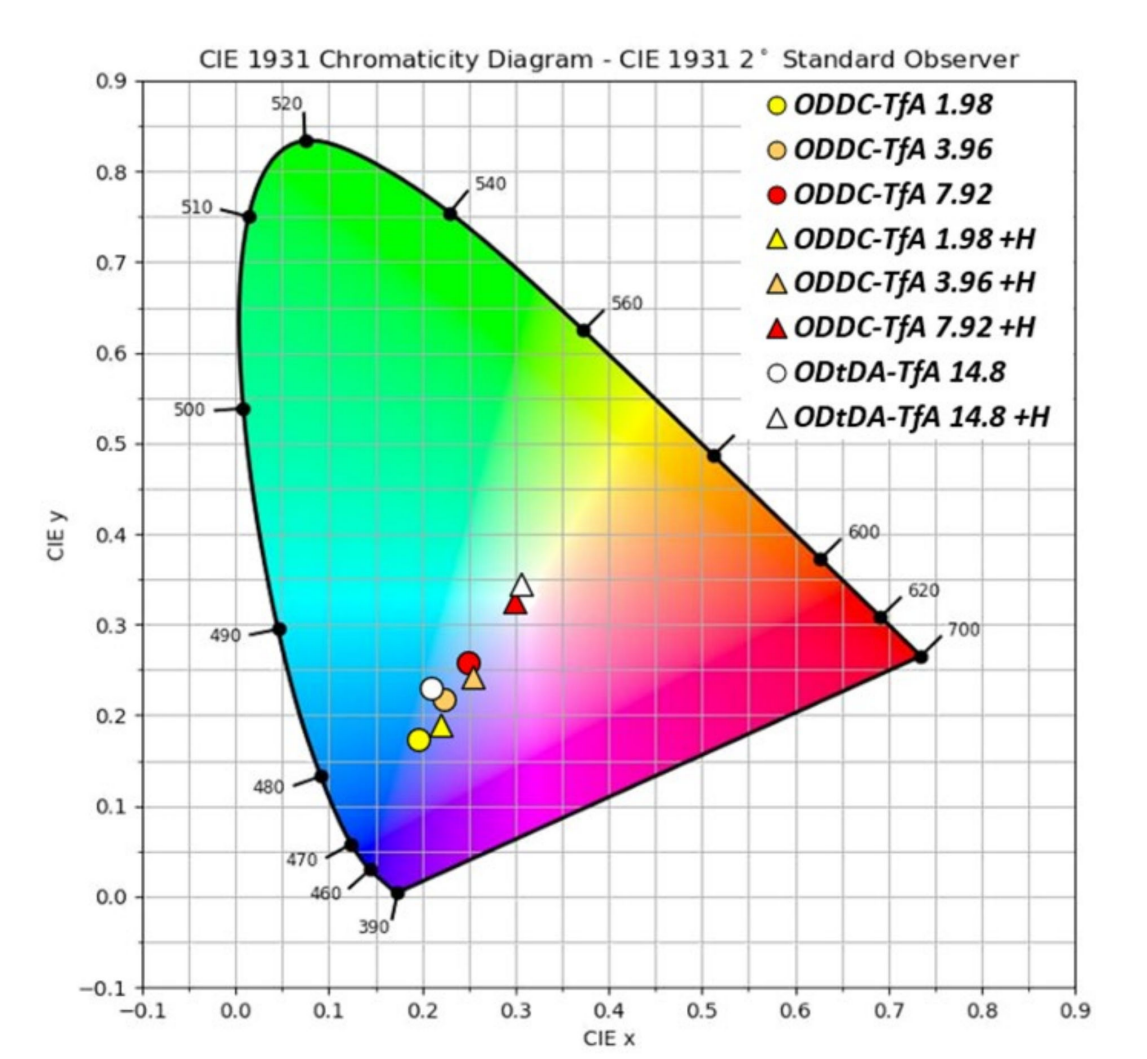
International Commission on Illumination (CIE) coordinates of the fluorescence colors of the PIs of ODDC-TfA and OD*t*DA-TfA, prepared with variable *r* values (*r* = 1.98, 3.96, 7.92, 14.8) and those doped with H_2_SO_4_ (0.5 ~ 1.0 eq., indicated by +H).

**Figure 6:**
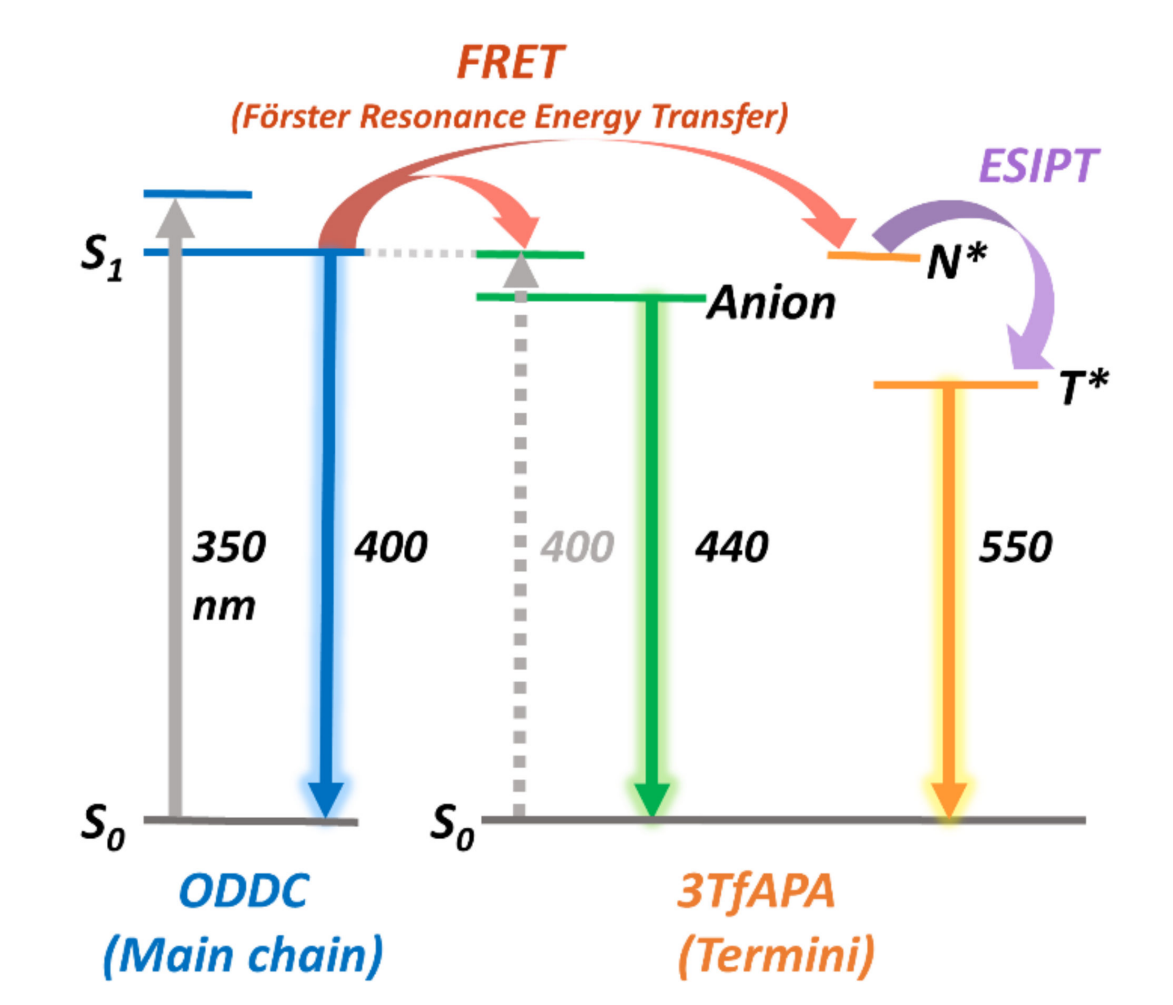
Photophysical processes for the end-capped PIs in the film state, illustrated by a schematic energy-state diagram.

Accordingly, the variations in the emission spectra of these PIs are visually displayed as the CIE chromaticity coordinates in Figure 5 and the values of the coordinates are listed in Table S1, while the photophysical processes in ODDC-TfA and H_2_SO_4_-doped ODDC-TfA are schematically shown in Figure 6. When the ODDC main chain is excited by UV light (*λ*_ex_ = 340–350 nm), a part of the excited energy is transferred to the 3TfAPA termini by FRET. Then, the excited T* form of 3TfAPA emits yellow fluorescence via ESIPT, while the excited anionic form simultaneously emits green fluorescence. Therefore, three fluorescent colors are observed by a single excitation wavelength for non-doped ODDC-TfA, whereas the anion emission is effectively suppressed by H_2_SO_4_-doping, which generates white-light luminescence.

Owing to the FRET from ODDC, 3TfAPA at the termini exhibited almost the same emission intensity as that of ODDC despite the small *r* value; pure white fluorescence was demonstrated by the H_2_SO_4_-doped ODDC-TfA with *r* = 7.92, located at (0.301, 0.327) in the CIE coordinates (Figure 4b). The estimated *Φ* value for the white-light emission was 9%. Consequently, a colorless and transparent end-capped PI that emits pure white fluorescence under UV light was successfully developed.

#### 3.2.2. Optical Properties of ODtDA-TfA

**Figure 7:**
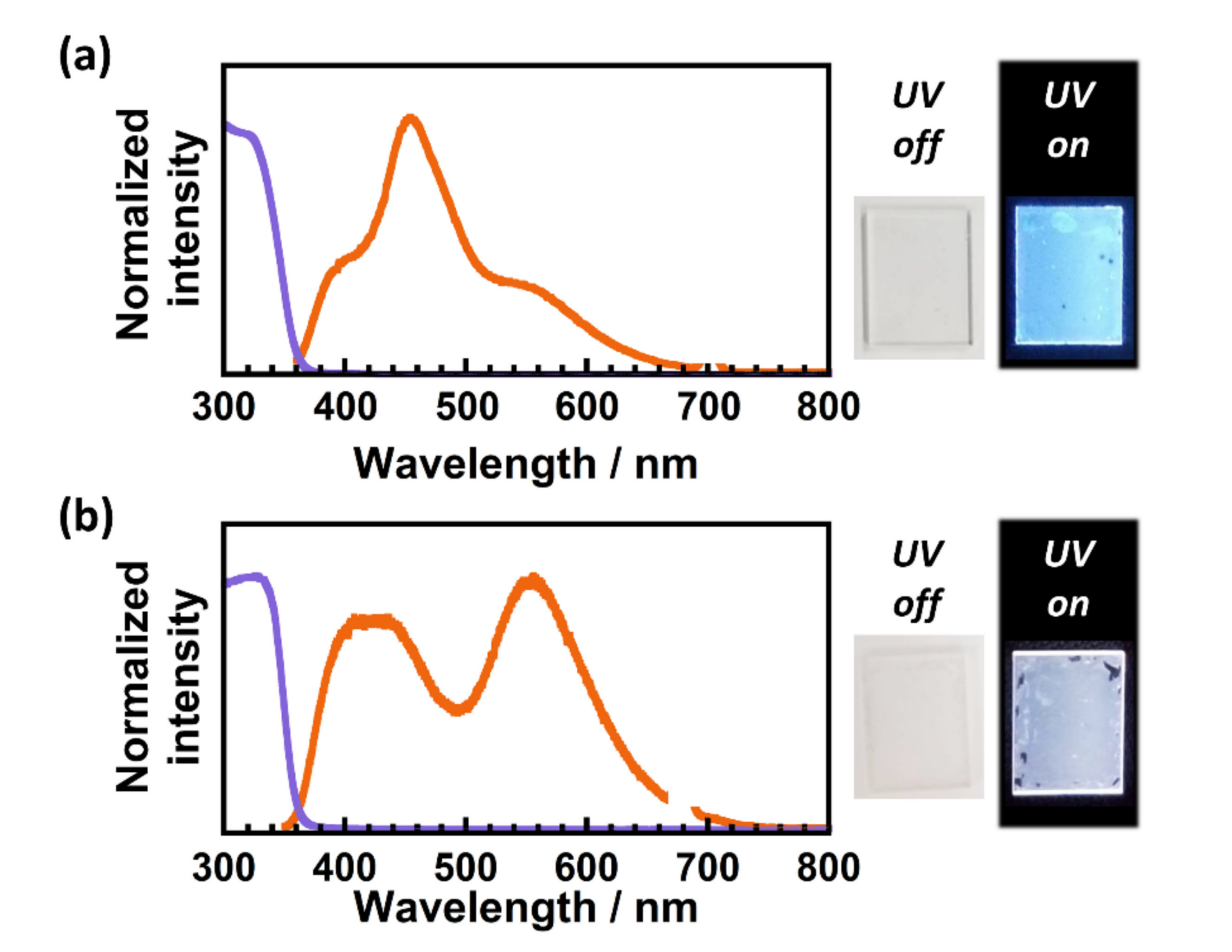
UV−vis absorption and emission spectra of (**a**) OD*t*DA-TfA and (**b**) OD*t*DA-TfA doped with H_2_SO_4_ (0.5 eq.), which were prepared with *r* = 14.8, and photographs of these films under white light (UV off) and UV (*λ* = 365 nm) irradiation (UV on).

To enhance the *Φ* (= 9%) of the white-light luminescence, *t*DACH was selected as the second diamine, as it consists of only one cyclohexyl ring. Due to the suppression of local molecular motion by the rigid diamine structure, OD*t*DA exhibited a *Φ* value (0.27) larger than that of ODDC (*Φ* = 0.11), by a factor of 2.5. In addition, when a small amount of H_2_SO_4_ was added to the PAA of OD*t*DA-TfA (H_2_SO_4_-doping), the green fluorescence emitted from the anionic form of 3TfAPA was effectively suppressed, similar to the case of ODDC-TfA. In Figure 7, the UV−vis absorption and emission spectra of the non-doped and H_2_SO_4_-doped (0.5 eq.) films of OD*t*DA-TfA with *r* = 14.8 are shown, along with photographs. Additionally, the values of *λ*_abs_, *λ*_ex_, *λ*_em_, *τ*, *E*_FRET_, and *Φ* are listed in Table 3. Time-resolved fluorescence decay curves are shown in Figure S6. Owing to the suppression of the anionic form and the enhanced T* fluorescence via FRET, pure white fluorescence was also achieved for the H_2_SO_4_-doped OD*t*DA-TfA with *r* = 14.8 (Figure 5 and Figure 7b), located at (0.307, 0.347) in the CIE coordinates. In addition, this *Φ* value of 0.16 is higher than that of H_2_SO_4_-doped ODDC-TfA by a factor of 1.66 due to the restricted local motion by OD*t*DA. In summary, bright white luminescent PI films working at room temperature were successfully prepared from a blue-fluorescent PI (ODDC) by end-capping with a yellow-fluorescent ESIPT anhydride with H_2_SO_4_ doping.

#### 3.2.3. Thermal Stability of the End-Capped PIs

The thermal stability of the white-light luminescent PI films is expected to be higher than 260–280 °C (soldering temperature), which is necessary for optical device fabrication. Figure S8 shows the thermogravimetric analysis (TGA) curves of the end-capped PIs, while their 5% weight-loss temperatures (*T*_d_^5^) are summarized in Table S2. The *T*_d_^5^s of the end-capped PIs range between 360 °C and 445 °C, indicating that all the PIs have sufficient thermal stability, thanks to their rigid PI main chain structures. The *T*_d_^5^ value decreased slightly with an increase in *r*. This result indicates that the heat resistance of the end-capped PIs is slightly lowered, since the chain length is shortened. In addition, some of the H_2_SO_4_-doped end-capped PIs showed a slight weight loss at approximately 337 °C, which corresponds to the boiling point of H_2_SO_4_.

## 4. Conclusions

An imide compound of 3TfAPI, forming an intramolecular hydrogen bond, was synthesized. Later, its photoluminescence properties were examined in a CHCl_3_ solution in the crystalline state and dispersed in a PMMA matrix. Bright yellow fluorescence via excited-state intramolecular proton transfer (ESIPT) was observed with large Stokes shifts (8.95 × 10^3^ < *ν* < 1.23 × 10^4^ cm^−1^) in all states. The fluorescence quantum yields (*Φ*) of 3TfAPI in the crystalline and PMMA were larger than that in solution, due to the suppression of local molecular motion and excited-state energy transfer by the bulky substituents. Additionally, an acid anhydride 3TfAPA with a chemical structure similar to that of 3TfAPI was synthesized, and a white-light luminescent polyimide (PI) (ODDC-TfA) was prepared by introducing 3TfAPA as the end-capping agent to a blue-fluorescent PI (ODPA/DCHM), with a molar ratio of 7.92. Anionic fluorescence was efficiently quenched with the addition of a small amount of H_2_SO_4_ to a precursor solution. White light fluorescence was achieved even with a small portion of 3TfAPA, owing to the Förster resonance energy transfer (FRET) from the ODDC main chain to the 3TfAPA termini. Moreover, another blue-fluorescent PI derived from *t*DACH diamine (OD*t*DA), whose *Φ* is 2.5 times as high as that of ODDC, was adopted as the blue fluorescent main chain. As a result, the non-radiative deactivation was further suppressed, and *Φ* was enhanced by a factor of 1.66 compared to ODDC-TfA. In summary, a novel white-light luminescent end-capped PI was successfully developed, based on FRET from the blue-fluorescent main chains and yellow ESIPT emission at the termini. This molecular design strategy for preparing colorless, transparent, thermally stable, and white-light-emitting PI materials can contribute to the miniaturization and weight reduction of optoelectronic devices.

## Figures and Tables

**Table 1:** Photoluminescence properties, absorption wavelengths (*λ*_abs_), emission wavelengths (*λ*_em_), Stokes shifts (*ν*), and quantum yields (*Φ*) of 3TfAPI dissolved in CHCl_3_, in the crystalline state and dispersed in PMMA matrix.

State	*λ*_abs_^a^/nm	*λ*_em_^a^/nm	*ν*/cm^−1^	*Φ*
CHCl_3_ solution	330	556	1.23 × 10^4^	0.17
Crystalline	365	542	8.95 × 10^3^	0.34
In PMMA matrix	333	549	1.18 × 10^4^	0.19

^a^ Experimental error of *λ* is estimated to be less than ± 1 nm.

**Table 2:** Photoluminescence properties, absorption wavelengths (*λ*_abs_), excitation/emission wavelengths (*λ*_ex_, *λ*_em_), fluorescence lifetimes at 400 nm (*τ*), FRET efficiencies (*E*_FRET_), and quantum yields (*Φ*) of ODDC and ODDC-TfA doped with 0.5 ~ 1.0 eq. H_2_SO_4_ prepared with variable *r* values (*r* = 1.98, 3.96, and 7.92).

	*r*	*λ*_abs_^a^/nm	*λ*_ex_^a^/nm	*λ*_em_^a^/nm	*τ*^b^/ns	*E*_FRET_	*Φ*
ODDC-TfA	0	<400	340	400	10.10	0.00	0.14
	1.98	<400	350	395 436 550	6.67	0.34	0.12
	3.96	<400	350	397 433 550	5.50	0.46	0.12
	7.92	<400	340	400 440 552	4.23	0.58	0.15
H_2_SO_4_-doped ODDC-TfA	1.98	<400	340	400 551	4.78	0.53	0.09
	3.96	<400	340	398 549	4.45	0.56	0.10
	7.92	<400	340	400 549	3.83	0.62	0.10

^a^ Experimental error of *λ* is estimated to be less than ±1 nm. ^b^ Experimental error of *τ* is estimated to be less than ±0.5 ns.

**Table 3:** Photoluminescence properties, absorption wavelengths (*λ*_abs_), excitation/emission wavelengths (*λ*_ex_, *λ*_em_), fluorescence lifetimes at 400 nm (*τ*), FRET efficiencies (*E*_FRET_), and quantum yields (*Φ*) of OD*t*DA and OD*t*DA-TfA with *r* = 14.8 without/with H_2_SO_4_ doping (0.5 eq.).

Polyimide	*r*	*λ*_abs_^a^/nm	*λ*_ex_ ^a^/nm	*λ*_em_ ^a^/nm	*τ* ^b^/ns	*E*_FRET_	*Φ*
OD*t*DA-TfA	0	<400	350	400	9.05	0.00	0.29
	14.8	<400	350	400 449 550	3.86	0.57	0.23
H_2_SO_4_-doped OD*t*DA-TfA	14.8	<400	350	420 555	3.95	0.56	0.16

^a^ The error of *λ* is estimated to be less than ±1 nm. ^b^ The error of τ is estimated to be less than ±0.5 ns.

## Supplementary Materials

The following are available online at https://www.mdpi.com/article/10.3390/polym13224050/s1, Scheme S1: Synthesis route of 3-trifluoroacetylamino-N-cyclohexylphthalimide (3TfAPI). Figure S1: 1H NMR spectrum of 3-nitro-N-cyclohexylphthalimide (S-2). Figure S2: 1H NMR spectrum of 3-amino-N-cyclohexylphthalimide (S-3). Figure S3: 1H NMR spectrum of 3-trifluoroacetylamino-N-cyclohexylphthalimide (3TfAPI). Scheme S2: Preparation scheme for polymethyl methacrylate (PMMA) dispersion thin film. Figure S4: 1H NMR spectrum of 3-aminophthalic acid hydrochloride (2). Figure S5: 1H NMR spectrum of 3-trifluoroacetylaminophthalic anhydride (3TfAPA). Figure S6: Time-resolved fluorescence decay curves for the end-capped PIs; (a) ODDC-TfA and (b) those doped with 0.5 ~ 1.0 eq. H2SO4 prepared with variable *r* values (*r* = 0, 1.98, 3.96, 7.98). (c) ODtDA-TfA and that doped with 0.5 eq. H2SO4 prepared with *r* = 0 and 14.8. Figure S7: Chemical structure of the anion form of ODDC-TfA. Table S1. Detailed International Commission on Illumination (CIE) coordinates for each end-capped PI (ODDC-TfA, ODtDA-TfA and those doped with H2SO4 (0.5 ~ 1.0 eq.) prepared with variable *r* values (*r* = 1.98, 3.96, 7.98, 14.8)). Figure S8: Thermogravimetric curves measured by thermogravimetric analysis (TGA) for the end-capped PIs (ODDC-TfA, ODtDA-TfA and those doped with 0.5 ~ 1.0 eq. H2SO4 prepared with variable *r* values (*r* = 1.98, 3.96, 7.98, 14.8)). Table S2: 5% weight reduction temperatures (Td5) for each end-capped PI (ODDC-TfA, ODtDA-TfA and those doped with H2SO4 (0.5 ~ 1.0 eq.) prepared with variable *r* values (*r* = 1.98, 3.96, 7.98, 14.8)).
