# Supplementary material for: Synthesis and Characterization of White-Light Luminescent End-Capped Polyimides Based on FRET and Excited State Intramolecular Proton Transfer
Source: Polymers (Basel). 2021 Nov 22;13(22):4050. doi: 10.3390/polym13224050 (PMC8621099; doi:10.3390/polym13224050)

checkCIF/PLATON report

Structure factors have been supplied for datablock(s) I

THIS REPORT IS FOR GUIDANCE ONLY. IF USED AS PART OF A REVIEW PROCEDURE FOR PUBLICATION, IT SHOULD NOT REPLACE THE EXPERTISE OF AN EXPERIENCED CRYSTALLOGRAPHIC REFEREE.

No syntax errors found. CIF dictionary Interpreting this report

Datablock: I

```
Bond precision:  C-C = 0.0021 A          Wavelength=0.71075

Cell:          a=5.1357(13)       b=10.360(3)       c=14.088(4)
               alpha=84.565(7)    beta=82.974(9)    gamma=89.991(9)
Temperature:   93 K
```

	Calculated	Reported
Volume	740.5(4)	740.5(3)
Space group	P -1	P -1
Hall group	-P 1	-P 1
Moiety formula	C16 H15 F3 N2 O3	C16 H15 F3 N2 O3
Sum formula	C16 H15 F3 N2 O3	C16 H15 F3 N2 O3
Mr	340.30	340.30
Dx,g cm-3	1.526	1.526
Z	2	2
Mu (mm-1)	0.131	0.131
F000	352.0	352.0
F000’	352.24	
h,k,lmax	6,13,18	6,13,18
Nref	3389	3362
Tmin,Tmax	0.994,0.996	0.816,0.996
Tmin’	0.983	

```
Correction method= # Reported T Limits: Tmin=0.816 Tmax=0.996
AbsCorr = MULTI-SCAN

Data completeness= 0.992          Theta(max)= 27.468

R(reflections)= 0.0401( 2126)     wR2(reflections)= 0.1007( 3362)

S = 0.898                  Npar= 220
```

The following ALERTS were generated. Each ALERT has the format
test-name_ALERT_alert-type_alert-level.
Click on the hyperlinks for more details of the test.

Alert level C

```
PLAT910_ALERT_3_C Missing # of FCF Reflection(s) Below Theta(Min).          5 Note
PLAT911_ALERT_3_C Missing FCF Refl Between Thmin & STh/L=    0.600         15 Report
PLAT913_ALERT_3_C Missing # of Very Strong Reflections in FCF ....          5 Note
PLAT934_ALERT_3_C Number of (Iobs-Icalc)/Sigma(W) > 10 Outliers ..          1 Check
```

Alert level G

```
CHEMS02_ALERT_1_G  Please check that you have entered the correct
            _publ_requested_category classification of your compound;
            FI or CI or EI for inorganic; FM or CM or EM for metal-organic;
            FO or CO or EO for organic.
            From the CIF: _publ_requested_category   CHOOSE FI FM FO CI CM CO or A
            From the CIF: _chemical_formula_sum :C16 H15 F3 N2 O3
PLAT912_ALERT_4_G Missing # of FCF Reflections Above STh/L=  0.600          6 Note
PLAT941_ALERT_3_G Average HKL Measurement Multiplicity ...........        2.7 Low
PLAT978_ALERT_2_G Number C-C Bonds with Positive Residual Density.          1 Info
```

```
   0 ALERT level A = Most likely a serious problem - resolve or explain
   0 ALERT level B = A potentially serious problem, consider carefully
   4 ALERT level C = Check. Ensure it is not caused by an omission or oversight
   4 ALERT level G = General information/check it is not something unexpected

   1 ALERT type 1 CIF construction/syntax error, inconsistent or missing data
   1 ALERT type 2 Indicator that the structure model may be wrong or deficient
   5 ALERT type 3 Indicator that the structure quality may be low
   1 ALERT type 4 Improvement, methodology, query or suggestion
   0 ALERT type 5 Informative message, check
```

Publication of your CIF

You should attempt to resolve as many as possible of the alerts in all categories. Often the minor alerts point to easily fixed oversights, errors and omissions in your CIF or refinement strategy, so attention to these fine details can be worthwhile. In order to resolve some of the more serious problems it may be necessary to carry out additional measurements or structure refinements. However, the nature of your study may justify the reported deviations from journal submission requirements and the more serious of these should be commented upon in the discussion or experimental section of a paper or in the "special_details" fields of the CIF. *checkCIF* was carefully designed to identify outliers and unusual parameters, but every test has its limitations and alerts that are not important in a particular case may appear. Conversely, the absence of alerts does not guarantee there are no aspects of the results needing attention. It is up to the individual to critically assess their own results and, if necessary, seek expert advice.

If you wish to submit your CIF for publication in Acta Crystallographica Section C or E, you should upload your CIF via the web. If you wish to submit your CIF for publication in IUCrData you should upload your CIF via the web. If your CIF is to form part of a submission to another IUCr journal, you will be asked, either during electronic submission or by the Co-editor handling your paper, to upload your CIF via our web site.

PLATON version of 13/07/2021; check.def file version of 13/07/2021

Datablock I - ellipsoid plot

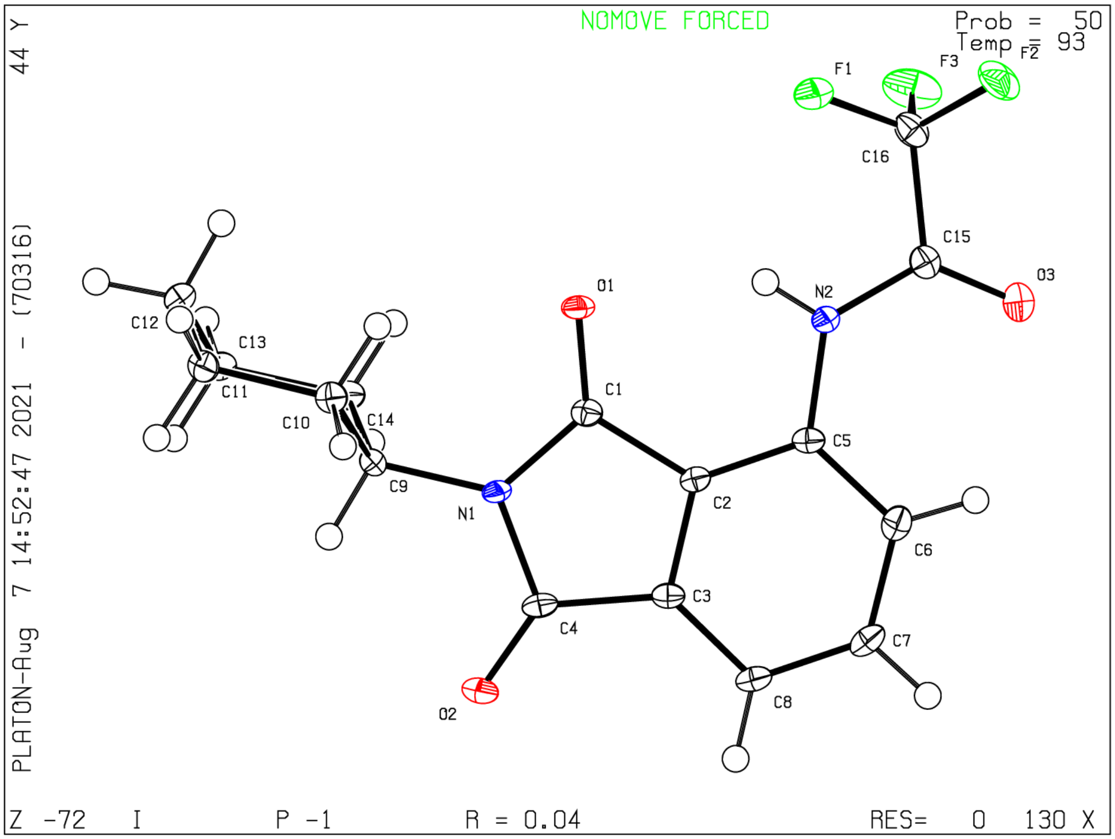

Supplement: Supplementary file 1 [file polymers-13-04050-s001.zip › checkcif.pdf]
