# Supplementary material for: Synthesis and Characterization of White-Light Luminescent End-Capped Polyimides Based on FRET and Excited State Intramolecular Proton Transfer
Source: Polymers (Basel). 2021 Nov 22;13(22):4050. doi: 10.3390/polym13224050 (PMC8621099; doi:10.3390/polym13224050)
Supplement: Supplementary file 1 [file polymers-13-04050-s001.zip › Revised Supporting Information_Polymers-1472129.pdf]

Supporting Information

Synthesis and Characterization of White-Light Luminescent End-Capped Polyimides Based on FRET and Excited State Intramolecular Proton Transfer

*Atsuko Tabuchi [a], Teruaki Hayakawa [b], Shigeki Kuwata [a], Ryohei Ishige [a], and Shinji Ando [a]**

[a] Department of Chemical Science & Engineering, Tokyo Institute of Technology, Ookayama 2-12-1, Meguro-ku, Tokyo 152-8552, Japan

[b] Department of Materials & Chemical Technology, Tokyo Institute of Technology, Ookayama 2-12-1, Meguro-ku, Tokyo 152-8552, Japan

1. Experimental Section

*Synthesis of 3-nitro-N−cyclohexylphthalimide (**S-2**).* Briefly, 3-nitrophthalic anhydride (5.79 g, 30 mmol) was refluxed in a mixture of cyclohexylamine (4.46 g, 45 mmol) and propionic acid (50 mL) for 6.5 h. After cooling to a temperature of 20 °C, a faint brown solid that was reprecipitated by excess water, was filtered and dried. White, needle-like crystals were obtained by recrystallization from ethanol (yield, 82%). For ^{1}H NMR (400 MHz, $CDCl_3$: δ [ppm] 1.25–1.39 (3H, br *m*, CH_2), 1.72–1.73 (3H, br *m*, CH_2), 1.86–1.87 (2H, br *m*, CH_2), 2.15–2.25 (2H, br *m*, CH_2), 4.15 (1H, *m*, N–CH), 7.90 (1H, *t*, J = 16.0 Hz, aryl), 8.08 (2H, *t*, J = 14.8 Hz, aryl) (**Figure S1**).

*Synthesis of 3-amino-N-cyclohexylphthalimide (**S-3**).* Compound **S-2** (4.13 g, 15 mmol) was dissolved in a mixture of ethanol (50 mL) and ethyl acetate (95 mL), followed by the addition of 10% palladium on carbon (Pd/C, wetted with ca. 55% water) (0.917 g). This mixture was subsequently stirred at 20 °C under a hydrogen atmosphere for 1 d. The reaction solution was passed through Celite 535 to remove Pd/C, and the filtrate was concentrated using a rotary evaporator. The resulting solid was recrystallized from ethanol, and vivid yellow plate-like

crystals were obtained (yield, 76%). For ^{1}H NMR (400 MHz, $CDCl_3$): δ [ppm] 1.24–1.41 (3H, br *m*, CH_2), 1.66–1.73 (3H, br *m*, CH_2), 1.84–1.87 (2H, br *m*, CH_2), 2.13–2.23 (2H, br *m*, CH_2), 4.04 (1H, *m*, N-CH), 5.22 (2H, br *s*, NH), 6.82 (1H, *d*, J = 8.4 Hz, aryl), 7.10 (1H, *d*, J = 7.6 Hz, aryl), 7.38 (1H, *t*, J = 15.6 Hz, aryl) (**Figure S2**).

*Synthesis of 3-trifluoroacetylamino-N-cyclohexylphthalimide (**3TfAPI**).* Compound **S-3** (0.50 g, 2.0 mmol) and sodium hydrogen carbonate (0.17g, 2.0 mmol) were added to 1,4-dioxane (4 mL), and trifluoroacetic anhydride (0.35 mL, 2.5 mmol) was added dropwise with stirring, after which the mixture was stirred for 30 min. The reaction solution with excess water was filtered and dried. White needle-like crystals were obtained by recrystallization from *n*-hexane (yield, 65%). For ^{1}H NMR (400 MHz, $CDCl_3$): δ [ppm] 1.25–1.42 (3H, br *m*, CH_2), 1.55–1.90 (5H, br *m*, CH_2), 2.09–2.22 (2H, br *m*, CH_2), 4.09 (1H, *m*, N-CH), 7.63 (1H, *d*, aryl), 7.75 (1H, *t* aryl), 8.68 (1H, *d*, aryl), 10.55 (1H, br *s*, NH) (**Figure S3**).
HRMS (FAB) m/z $[M + H]^+$ calcd for $C_{16}H_{15}N_2O_3F_3$ 341.1113; found 341.1119.
Anal. Calced for $C_{16}H_{15}N_2O_3F_3$: C, 56.47; H, 4.44; N, 8.23; O, 14.10; F, 16.75. Found: C, 56.85; H, 4.19; N, 8.23; O+F, 30.73.

2. Figures and Table

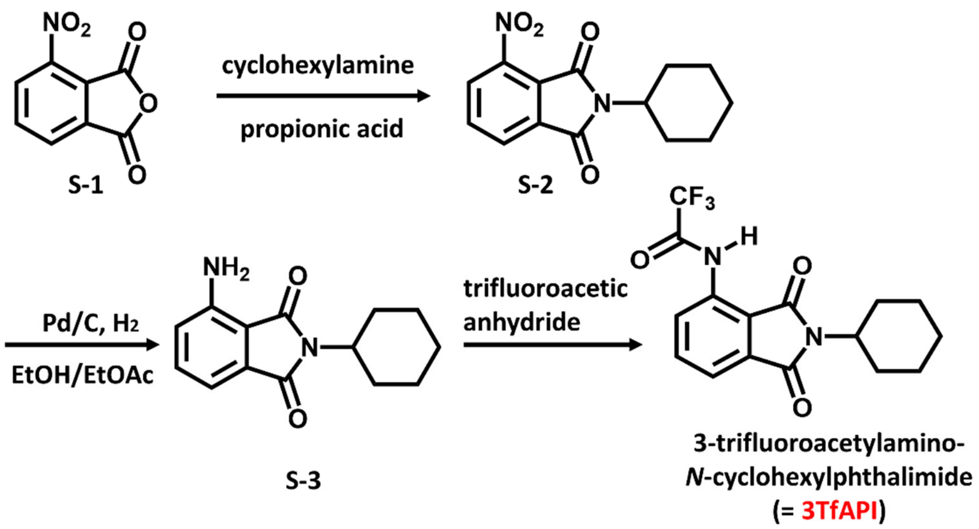

Scheme S1. Synthesis route of 3-trifluoroacetylamino-*N*-cyclohexylphthalimide (**3TfAPI**).

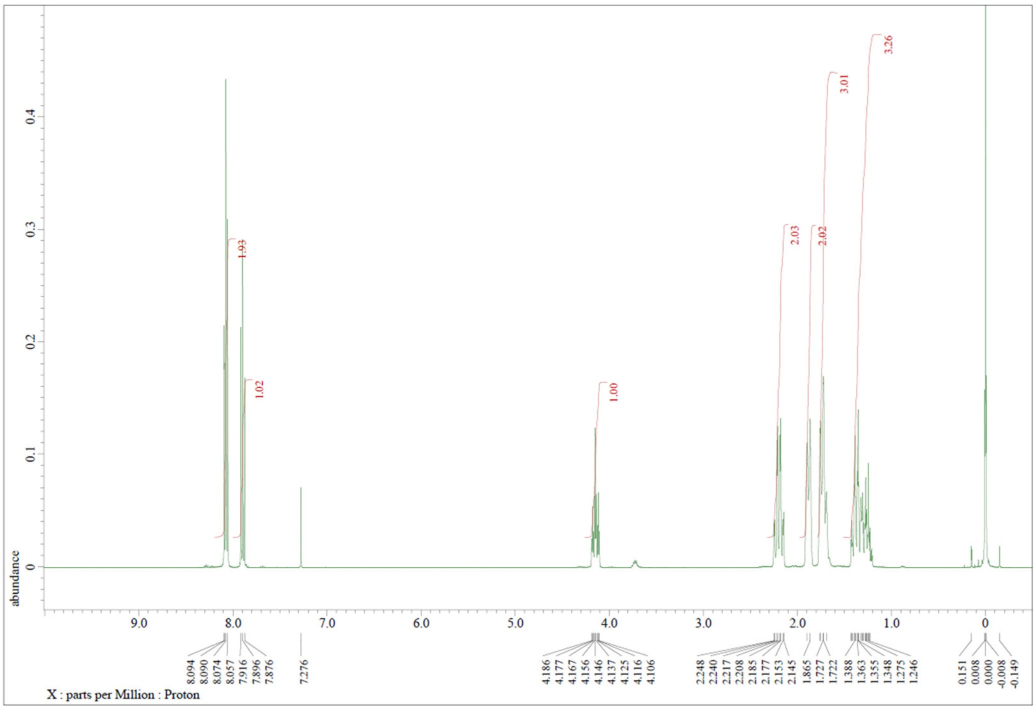

Figure S1. ^{1}H NMR spectrum of 3-nitro-*N*-cyclohexylphthalimide (**S-2**).

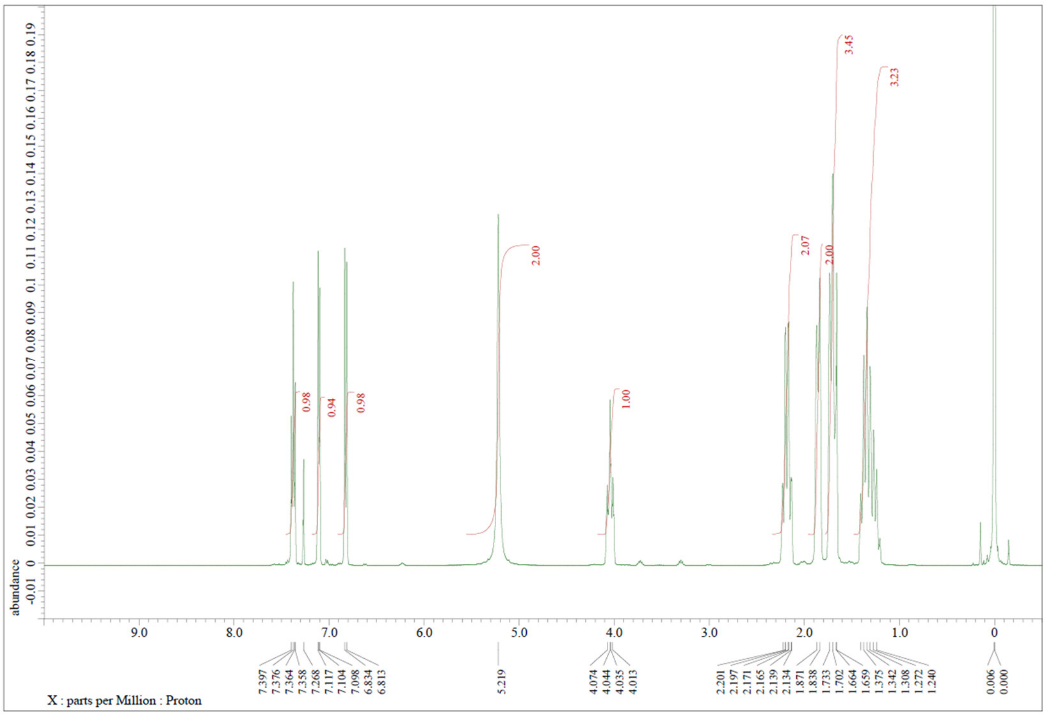

Figure S2. ^{1}H NMR spectrum of 3-amino-*N*-cyclohexylphthalimide (**S-3**).

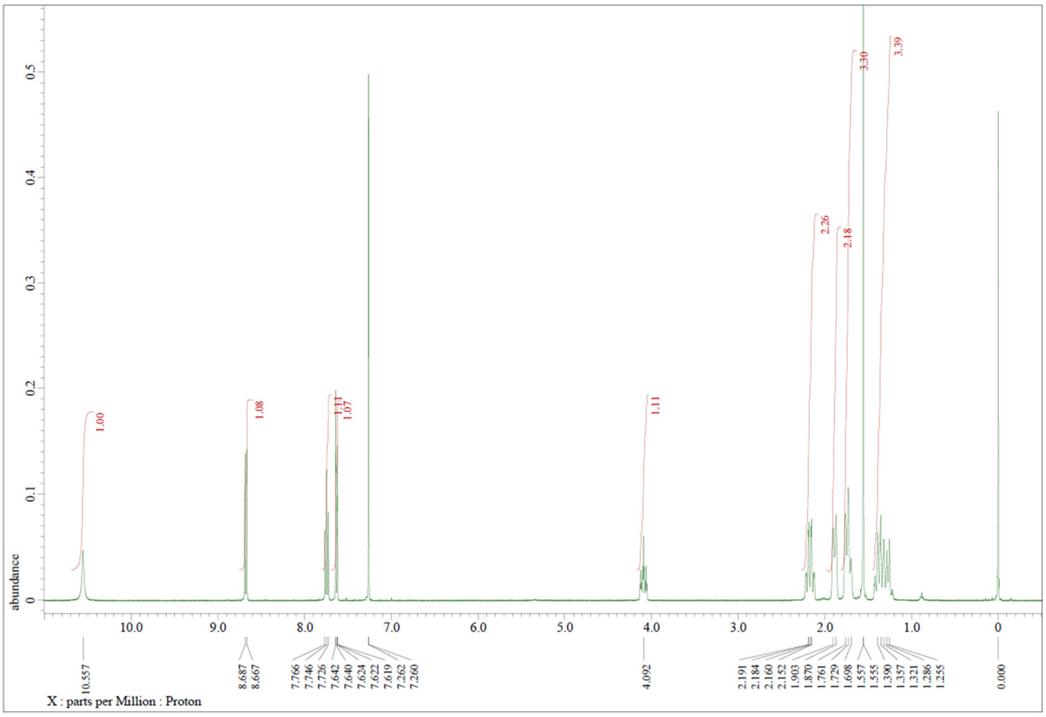

Figure S3. ^{1}H NMR spectrum of 3-trifluoroacetylamino-*N*-cyclohexylphthalimide (3TfAPI).

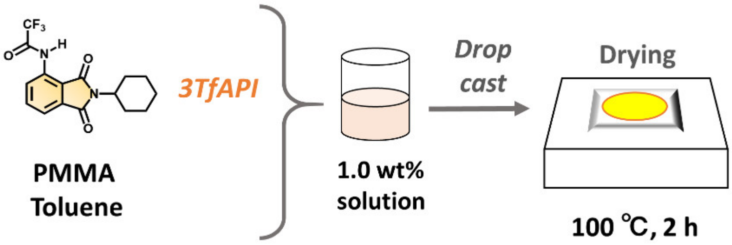

Scheme S2. Preparation scheme for polymethyl methacrylate (PMMA) dispersion thin film.

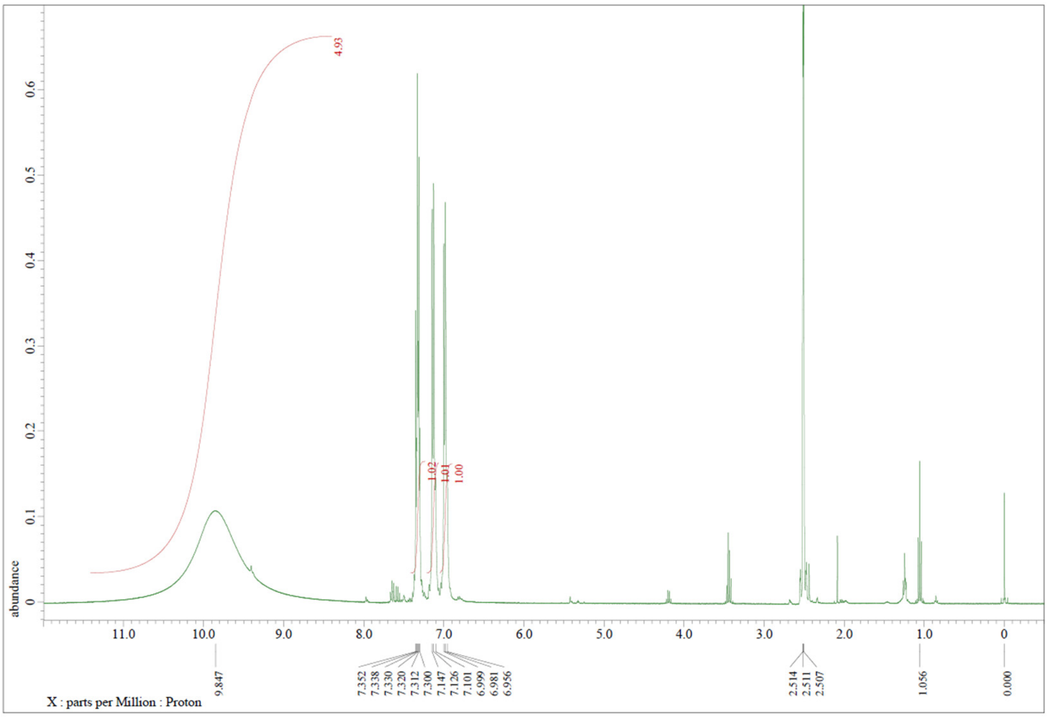

Figure S4. ^{1}H NMR spectrum of 3-aminophthalic acid hydrochloride (**2**).

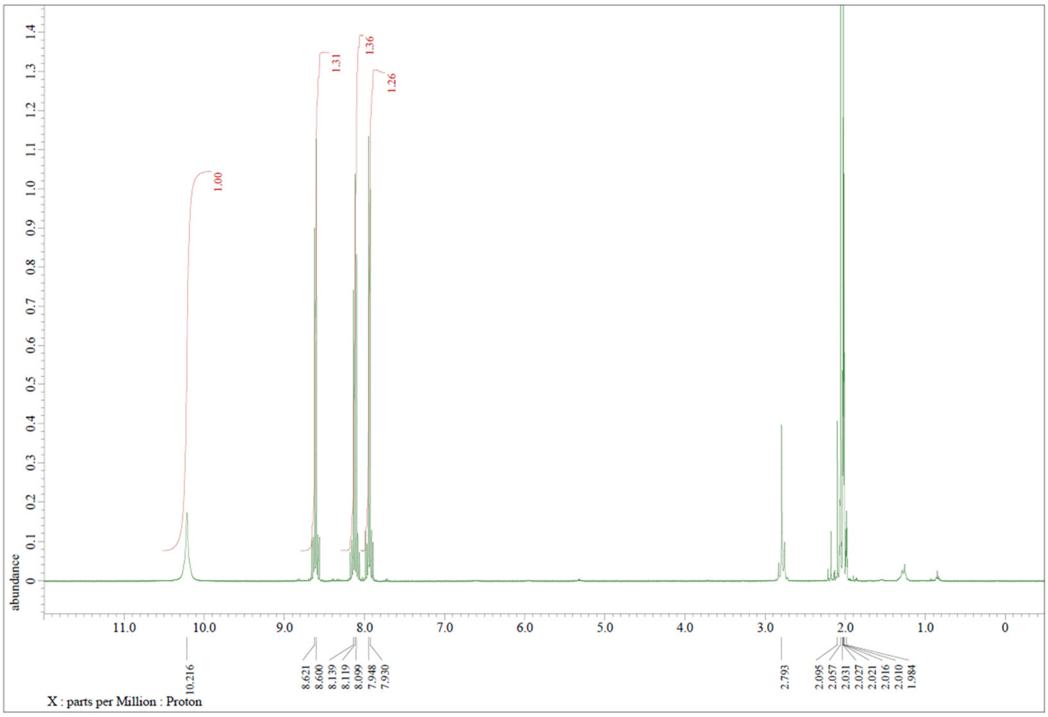

Figure S5. ^{1}H NMR spectrum of 3-trifluoroacetylaminophthalic anhydride (3TfAPA).

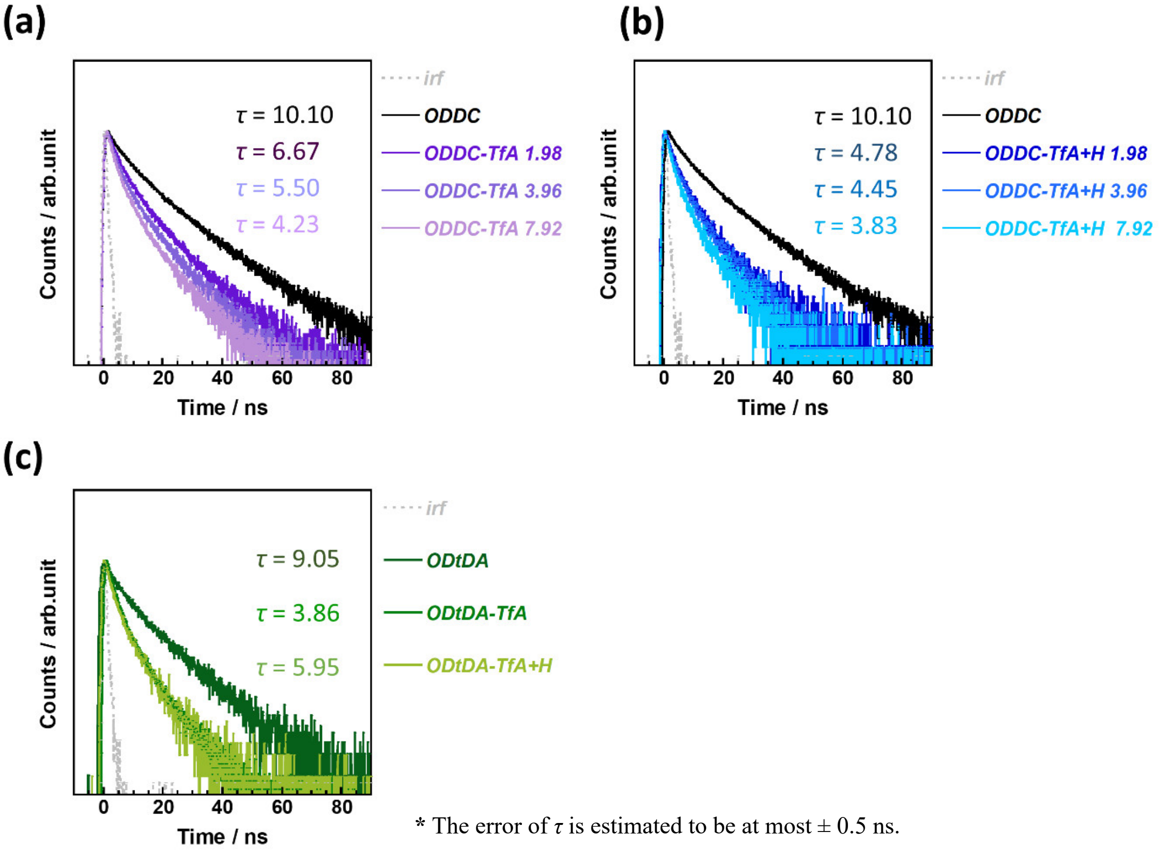

* The error of τ is estimated to be at most ± 0.5 ns.

Figure S6. Time-resolved fluorescence decay curves for the end-capped PIs; (a) **ODDC-TfA** and (b) those doped with 0.5 ~ 1.0 eq. H_2SO_4 prepared with variable r values (r = 0, 1.98, 3.96, 7.98). (c) **OD*t*DA-TfA** and that doped with 0.5 eq. H_2SO_4 prepared with r = 0 and 14.8.

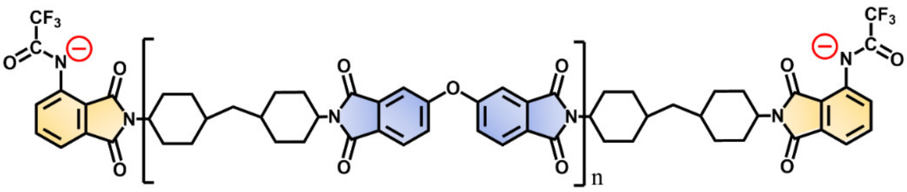

Figure S7. Chemical structure of the anion form of **ODDC-TfA**.

Table S1. Detailed International Commission on Illumination (CIE) coordinates for each end-capped PI (**ODDC-TfA**, **OD*t*DA-TfA** and those doped with H_2SO_4 (0.5 ~ 1.0 eq.) prepared with variable *r* values (*r* = 1.98, 3.96, 7.98, 14.8)).

Polyimide (PI)	*r*	CIE coordinates
**ODDC-TfA**	1.98	(0.198, 0.174)
	3.96	(0.225, 0.218)
	7.92	(0.252, 0.258)
**H_2SO_4- doped ODDC-TfA (0.5~1.0 eq.)**	1.98	(0.221, 0.192)
	3.96	(0.255, 0.244)
	7.92	(0.301, 0.327)
**OD*t*DA-TfA**	14.8	(0.212, 0.230)
**H_2SO_4-doped OD*t*DA-TfA (0.5 eq.)**	14.8	(0.307, 0.347)

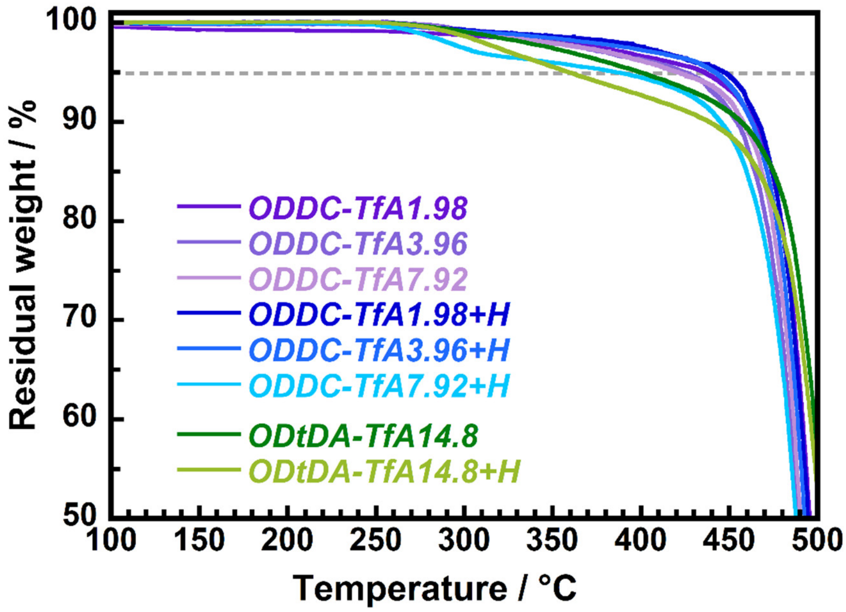

Figure S8. Thermogravimetric curves measured by thermogravimetric analysis (TGA) for the end-capped PIs (**ODDC-TfA**, **OD*t*DA-TfA** and those doped with 0.5 ~ 1.0 eq. H_2SO_4 prepared with variable *r* values (*r* = 1.98, 3.96, 7.98, 14.8)).

Table S2. 5% weight loss temperatures (***T*****d**[5]) for each end-capped PI (**ODDC-TfA**, **OD*t*DA-TfA** and those doped with H_2SO_4 (0.5 ~ 1.0 eq.) prepared with variable *r* values (*r* = 1.98, 3.96, 7.98, 14.8)).

Polyimides (PIs)	*r*	T_d^5 (°C)
**ODDC-TfA**	1.98	436
	3.96	425
	7.92	423
**H_2SO_4- doped ODDC-TfA (0.5~1.0 eq.)**	1.98	447
	3.96	442
	7.92	387
**OD*t*DA-TfA**	14.8	400
**H_2SO_4-doped OD*t*DA-TfA (0.5 eq.)**	14.8	360
